# Identification of the *S*-transferase like superfamily bacillithiol transferases encoded by *Bacillus subtilis*

**DOI:** 10.1371/journal.pone.0192977

**Published:** 2018-02-16

**Authors:** Varahenage R. Perera, John D. Lapek, Gerald L. Newton, David J. Gonzalez, Kit Pogliano

**Affiliations:** 1 Division of Biological Sciences, University of California, San Diego, La Jolla, CA, United States of America; 2 Department of Pharmacology and Pharmacy, School of Medicine, University of California, San Diego, La Jolla, CA, United States of America; Centre National de la Recherche Scientifique, Aix-Marseille Université, FRANCE

## Abstract

Bacillithiol is a low molecular weight thiol found in Firmicutes that is analogous to glutathione, which is absent in these bacteria. Bacillithiol transferases catalyze the transfer of bacillithiol to various substrates. The *S*-transferase-like (STL) superfamily contains over 30,000 putative members, including bacillithiol transferases. Proteins in this family are extremely divergent and are related by structural rather than sequence similarity, leaving it unclear if all share the same biochemical activity. *Bacillus subtilis* encodes eight predicted STL superfamily members, only one of which has been shown to be a bacillithiol transferase. Here we find that the seven remaining proteins show varying levels of metal dependent bacillithiol transferase activity. We have renamed the eight enzymes BstA-H. Mass spectrometry and gene expression studies revealed that all of the enzymes are produced to varying levels during growth and sporulation, with BstB and BstE being the most abundant and BstF and BstH being the least abundant. Interestingly, several bacillithiol transferases are induced in the mother cell during sporulation. A strain lacking all eight bacillithiol transferases showed normal growth in the presence of stressors that adversely affect growth of bacillithiol-deficient strains, such as paraquat and CdCl_2_. Thus, the STL bacillithiol transferases represent a new group of proteins that play currently unknown, but potentially significant roles in bacillithiol-dependent reactions. We conclude that these enzymes are highly divergent, perhaps to cope with an equally diverse array of endogenous or exogenous toxic metabolites and oxidants.

## Introduction

Low molecular weight thiols (LMWTs) are small, non-protein organosulfur molecules that play many roles in the cell. Notably, they serve as cytoplasmic redox buffers, protect cysteine residues from overoxidation, and they can detoxify harmful molecules that are either produced intracellularly and/or encountered from the extracellular environment[1,2]. The major LMWT produced in both eukaryotes and Gram-negative bacteria is glutathione, and the enzymes that catalyze the nucleophilic attack of glutathione to target substrates are glutathione transferases (GSTs,[3]). Thiol transferases are usually associated with conjugating thiols to small molecules in detoxification reactions. However, some thiol transferases have been found to add thiols to protein substrates. GST Pi, a class of cytosolic human GSTs, have been shown to glutathionylate proteins *in vivo* in humans[4], and *in vitro*[5]. Eukaryotic GSTs have garnered much attention due to their implications in mammalian disease states such as Parkinson’s and Alzheimer’s disease[3,6,7] and their ability to detoxify electrophilic toxins such as carcinogens[8] and cancer drugs[9], while bacterial GSTs have been implicated in antibiotic resistance, isomerization reactions, and bioremediation[10]. Thus, glutathione and GSTs play a number of physiological roles and are central to several biochemical pathways.

It has recently been recognized that some bacteria use alternate low molecular weight thiols rather than glutathione, including mycothiol, which is produced by Actinobacteria such as *Mycobacterium smegmatis*, and bacillithiol, which is produced by Firmicutes such as *Bacillus subtilis* and *Staphylococcus aureus*. The roles of these thiols are less well understood than those of glutathione. Mycothiol buffers the cytoplasm, detoxifies electrophiles[11], and, via dedicated mycothiol transferases, it participates in the isomerization of malylpyruvate to fumarylpyruvate[12] and forming new sulfur-carbon bonds during biosynthesis of the secondary metabolites lincomycin A[13] and ergothioneine[14]. Bacillithiol been found to detoxify the cell wall biogenesis inhibitor fosfomycin[15], to protect cysteine residues during NaOCl stress [16,17], and to be involved in Fe(S) cluster metabolism[18,19].

Thus far, two families of bacillithiol transferases that lack sequence and structural similarity have been identified in the Firmicutes. The first bacillithiol transferase identified in *B*. *subtilis* was FosB, which belongs the vicinal oxygen chelate (VOC) superfamily [15,20]. The FosB bacillithiol transferase catalyzes bacillithiol-dependent detoxification of fosfomycin, and mutants in *fosB* and bacillithiol biosynthesis genes are sensitive to the antibiotic[15,21]. The second bacillithiol transferase to be identified in *B*. *subtilis* was BstA (YfiT; [22]), which is a member of the *S*-transferase-like (STL, formerly DinB/YfiT) superfamily;[23]). The STL superfamily is large, containing more than 30,000 predicted enzymes from both Gram-negative and Gram-positive bacteria. It is a remarkably diverse family, with membership defined not by primary sequence similarity, but rather by the ability to fold into similar structures as predicted by hidden Markov models ([24], http://supfam.org/SUPERFAMILY/). There is wide variation in the number of STL family members per genome: while some species encode only a single STL family member, others encode as many as 30. For example, *S*. *aureus* encodes one bacillithiol transferase, while *B*. *subtilis* encodes eight distantly related family members, with only two of the enzymes sharing >30% sequence similarity and the rest sharing only predicted structural similarity. However, it remains unclear if all the members of the STL superfamily are bacillithiol transferases, because thus far, only two of the STL proteins have been characterized and confirmed to be active bacillithiol transferases: *B*. *subtilis* BstA[22] and *S*. *aureus* BstA[23].

Given the critical role of glutathione transferases in eukaryotic cells[3] and the role of FosB in detoxification of the antibiotic fosfomycin[15,21], we hypothesized that characterization of this new family of proteins might provide insight into the role of bacillithiol in the Firmicutes and potentially allow identification of novel antibiotic and oxidative stress resistance pathways and possibly even new drug targets. This is especially important for pathogenic bacteria, such as *B*. *anthracis*, and industrially relevant bacteria, such as *B*. *subtilis*, that encode several putative STL bacillithiol transferases. However, identifying the physiological roles and natural substrates of the STL bacillithiol transferases will be difficult without additional information about these enzymes.

To address this limitation, we chose to characterize the seven remaining putative bacillithiol transferases in *B*. subtilis, reasoning that they would provide an ideal system for testing if all the members of this diverse protein family in this species are bacillithiol transferases, and if so, that *B*. *subtilis* might be an ideal organism for investigating their role. *B*. *subtilis* is genetically and biochemically tractable soil-dwelling organism with several well characterized developmental states, such as sporulation. It lives in complex, mixed species communities, in an environment awash with antimicrobial toxins and metabolic byproducts, and it also produces a variety of secondary metabolites such as the polyketide bacillaene, which arrests protein translation in *E*. *coli* [25]. *B*. *subtilis* is also used in industrial applications as a biological control agent for agricultural applications[26–28] and as a host for the overproduction of secreted proteins[29,30]. It is plausible that bacillithiol transferases might be necessary for survival in these complex conditions, either by directly contributing to antibiotic resistance mechanisms or biosynthetic pathways, or to other resistance mechanisms necessary to survive in harsh environments. Indeed conjugates of the analogous low molecular weight thiol from Actinobacteria, mycothiol, to natural products and antibiotics have been isolated from industrial fermentation cultures of actinomycetes[11].

In this study, we describe the activity and expression of the *B*. *subtilis* STL bacillithiol transferases. Bioinformatic studies of the eight *B*. *subtilis* STL proteins revealed that most are conserved in related *Bacillus spp*, and that all but one is also found in the pathogen *Bacillus anthracis*, indicating evolutionary pressure to retain the proteins. We have confirmed that the seven previously uncharacterized STL proteins encoded by *B*. *subtilis* are active bacillithiol transferases. We characterized the expression patterns of the proteins during different phases of growth and sporulation, and identified proteins that are *S*-bacillithiolated during growth. We constructed a strain lacking all eight bacillithiol transferases but thus far, have been unable to find a clear phenotype associated with this strain. Indeed, the strain lacks the phenotypes found in mutants that cannot synthesize bacillithiol, indicating that the bacillithiol transferases are not involved in the detoxification of those molecules. Thus, our studies demonstrate that all eight of the STL proteins encoded by *B*. *subtilis* are active bacillithiol transferases, but their role remains unclear.

## Materials and methods

### Strains and culture conditions

All the strains used in this study (Table G in S1 File) are derivatives of *B*. *subtilis* PY79, with the exception of the CU1065-derived strains obtained from the John Helmann lab. Heat resistant spore titers were assayed on cultures grown and sporulated in DSM broth [31] for 24 h at 37°C. Cultures were heated at 80°C for 20 min, serially diluted, and plated on LB. Spore titers were calculated based on colony counts. Sporulation for microscopy and mass spectrometry experiments were induced by growing cells in ¼ LB and re-suspension [32,33] at 37°C. Plasmid constructions were performed in *E*. *coli* Top10. The *B*. *subtilis* strain KP1302 was used for cloning purposes only. All cultures were grown in LB medium or Spizizen’s minimal medium at 37°C. Spizizen’s minimal medium was supplemented 0.05% casamino acids (Difco) and 1x trace metal solution was added (100x trace metal solution contains 62 mM MgCl_2_, 5 mM CaCl_2_, 1.3 mM ZnCl_2_, 500 μM MnCl_2_, 250 μM CuCl_2_, 460 μM CoCl_2_, 250μM Na_2_MoO_4_). Metals were purchased from Sigma Aldrich and Fisher Scientific.

### Plasmid and strain construction

A list of plasmids, strains and oligonucleotides used in this study can be found in Tables F-H in S1 File. All strains were constructed using either NEBuilder (New England Biolabs, Inc) or Gibson Assembly (Synthetic Genomics, Inc, [34,35]). Either Velocity (Bioline) or Phusion (New England Biolabs, Inc) DNA polymerase was used to PCR amplify all fragments. The integration vector pDG1730 [36] was used to insert the promoter region (>400 base pairs upstream of the gene) of each *bst* fused to the *sfGFP* gene at the *amyE* locus for the bacillithiol transferase promoter fusion strains. After strain construction, the *amyE* insert was amplified and sequenced to ensure that mutations had not been introduced into the fragments.

For the single *bstA-H* deletion strains, an antibiotic cassette, the region upstream of the gene and the region downstream of the gene were assembled to form the final plasmid. The antibiotic cassette was amplified from pJLG38[37], which contains a kanamycin cassette flanked by *loxP* sites. Different versions of this plasmid replacing the kanamycin cassette with chloramphenicol, erythromycin, tetracycline, and spectinomycin were constructed (Table F, Table G and Table H in S1 File). The chloramphenicol, spectinomycin, and erythromycin antibiotic markers for the deletion strains were amplified from integration vectors obtained from the *Bacillus* Genetic Stock Center (http://bgsc.org), while the tetracycline marker was amplified from the *Bacillus megaterium* vector pWH1520 (http://www.mobitec.com). The *loxP* sites flanking each antibiotic resistance gene were arranged so that after insertion into the chromosome at the *bst* genes, they would be in the same orientation around the genome to avoid Cre-mediated inversions (*yfiT*, *yuaE*, and *ykkA* are in the opposite orientation of the other *bst* genes). Single mutations were confirmed by sequencing to ensure that mutations had not been introduced into the genome.

The Δ*bstA-H* octuple mutant was constructed by consecutively transforming single *bst* mutants, each with their antibiotic marker flanked by *loxP* sites, into PY79 derivative EBS42. This strain contains *cre* under the control of the *spoIIR* promoter, allowing removing lf the antibiotic resistance markers. Marker removal was performed by sporulating cells overnight at 37°C in DSM, heating the culture for 20 min at 80°C to kill vegetative cells, and plating cells out on LB. Cells were patched on antibiotic plates to check for the loss of antibiotic resistance, and each strain was tested by PCR to confirm the absence of the markers. After deleting *dinB*, *yizA*, *yrdA*, *yuaE*, *yfiT*, and *ykkA*, the *sacA* locus containing *P*_*spoIIR*_*-cre* was restored by transforming a plasmid containing the intact *sacA* gene linked to a *tet* marker. We selected on tetracycline plates the next day and patched cells on spectinomycin plates to check for the loss of spectinomycin resistance. The final two *bst* mutants *ΔyisT*::*loxP*-*cm-loxP* and *ΔyjoA*::*loxP-spec-loxP* were transformed to construct the final *ΔbstA-H* mutant.

### Phylogenetic analysis

**Fig 1:**
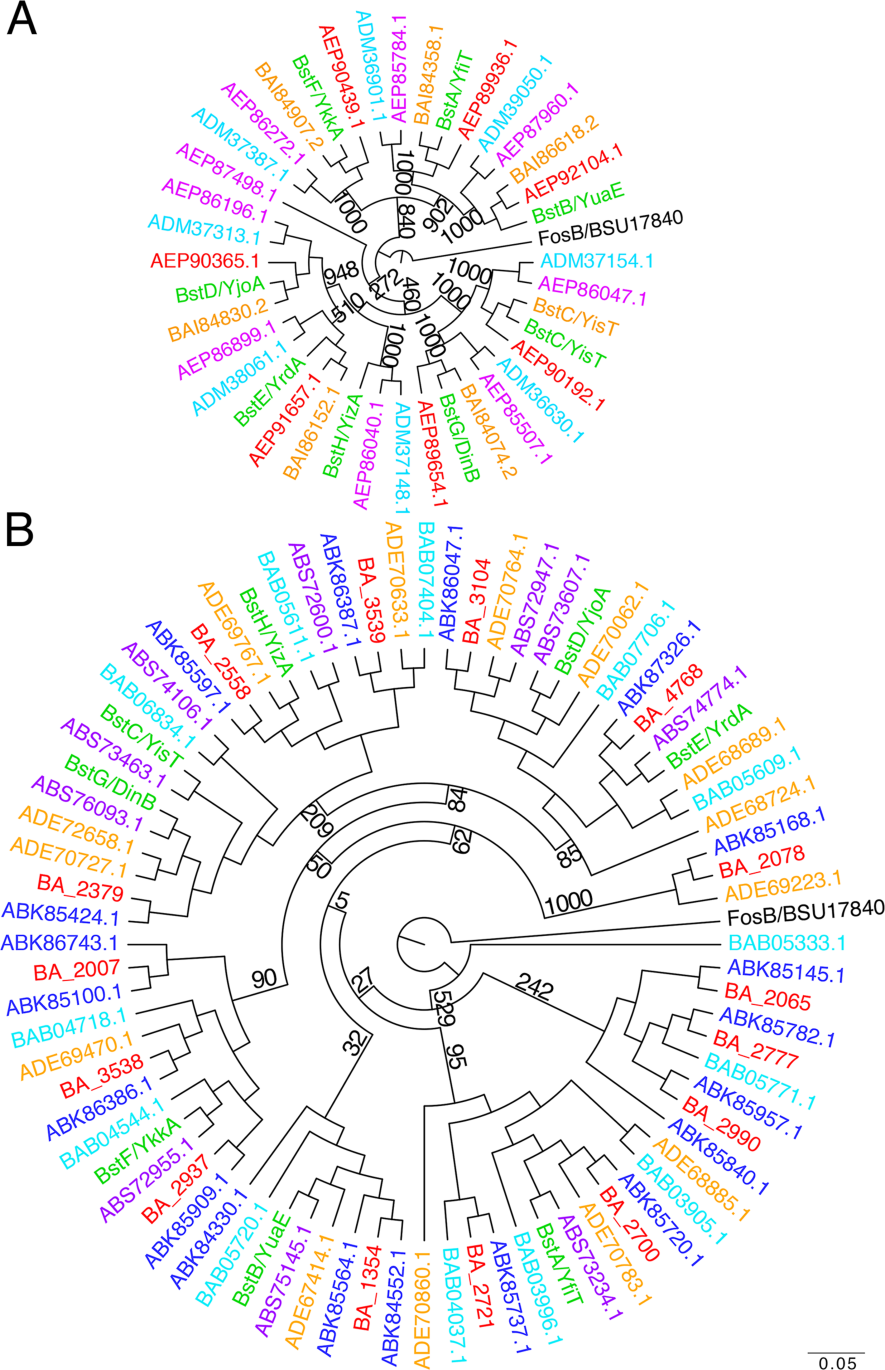
Phylogenetic analysis of STL bacillithiol transferases in *Bacillus subtilis* strains and *Bacillus spp*. Proteins are listed using NCBI locus tags. Bootstrap values are shown for selected branches and correspond to confidence levels. A) Black: *B*. *subtilis* 168 FosB (outgroup), green: *Bacillus subtilis* 168 proteins, orange: *Bacillus subtilis subsp*. *natto* BEST195 proteins, magenta: *Bacillus subtilis subsp*. *spizizenii* TU-B-10 proteins, cyan: *Bacillus subtilis subsp*. *spizizenii* str. W23 proteins, red: *Bacillus subtilis subsp*. *subtilis* str. RO-NN-1 proteins See Table A in S1 File for additional information. B) Black: *B*. *subtilis* 168 FosB (outgroup), green: *Bacillus subtilis* 168 proteins, orange: *Bacillus megaterium* QM B1551 proteins, purple: *Bacillus amyloliquefaciens subsp*. *plantarum str*. *FZB42* proteins, cyan: *Bacillus halodurans* C-125 proteins, red: *Bacillus anthracis str*. *Ames*, blue: *Bacillus thuringiensis* str. Al Hakam. See Table B in S1 File for additional information.

The first phylogenetic tree (Fig 1A) contains STL superfamily members from five different strains of *Bacillus subtilis*: the parent strain of PY79 *B*. *subtilis* 168 (NC_000964.3), *B*. *subtilis subsp*. *natto* BEST195 (NC_017196.2), *B*. *subtilis subsp*. *spizizenii* TU-B-10 (NC_016047.1), *B*. *subtilis subsp*. *spizizenii* str. W23 (NC_014479.1), *B*. *subtilis subsp*. *subtilis* str. RO-NN-1 (NC_017195.1). See Table A in S1 File for additional information on the individual proteins found in each strain. The second tree contains STL superfamily members from other *Bacillus* species, including *B*. *subtilis* 168 FosB, *B*. *subtilis* 168 proteins *B*. *megaterium* QM B1551 proteins, *B*. *amyloliquefaciens subsp*. *plantarum str*. *FZB42* proteins, *Bacillus halodurans* C-125 proteins, *Bacillus anthracis str*. *Ames*, *Bacillus thuringiensis* str. Al Hakam. See Table B in S1 File for additional information.

ClustalX was used to align sequences using the PAM series and to construct phylogenetic trees. Trees were generated by the neighbor-joining clustering method with 1000 bootstrap trials and were visualized using FigTree v1.4.0 (http://tree.bio.ed.ac.uk/software/figtree/). *B*. *subtilis* FosB was used as an outgroup in both trees. Similar trees were obtained regardless of the method used.

### Bacillithiol transferase recombinant protein cloning, purification, and assessment of biochemical activity

The protein sequences of the putative *B*. *subtilis* bacillithiol transferases YuaE, YisT, YjoA, YrdA, YkkA, DinB, and YizA were codon optimized for expression in *E*. *coli* and synthesized by GenScript (Piscataway, New Jersey, USA) and cloned into the pET28a+ expression vector to append the N-terminal His_6_ tag sequence to the *bst* sequences, giving a total of 20 additional amino acids at the N-terminus (Genscript, New Jersey). As was the case for *S*. *aureus* BstA, the recombinant proteins appeared to be toxic to many commercial *E*. *coli* expression hosts, including BL21(DE3), and it produced inclusion bodies after induction with IPTG. We therefore expressed the proteins in *E*. *coli* strain C41(DE3), which was selected to allow expression of toxic proteins[38]. One liter of Luria Broth containing kanamycin (50 μg/mL) was inoculated with cells and grown to late exponential phase. Cells were induced with 1 mM IPTG for 3 hours at 30°C, and cells were lysed and the proteins were purified on a Zn^2+^ resin as previously described[23]. Protein concentration was estimated using the following extinction coefficients (assuming all cysteine residues are reduced): BstB = 0.776 mg mL^− 1^, BstC = 1.334 mg mL^− 1^, BstD = 0.373 mg mL^− 1^, BstE = 1.517 mg mL^− 1^, BstF = 1.608 mg mL^− 1^, BstG = 1.106 mg mL^− 1^, and BstH = 2.0 mg mL^− 1^ (ExPASy ProtParam tool; http://ca.expasy.org). The purified proteins were analyzed on an SDS PAGE gel (Figure B in S1 File). Proteins were analyzed on BOLT 4–12% Bis-Tris SDS PAGE gels using BOLT MES SDS running buffer (Invitrogen; Figure B in S1 File). The protein standard used was SeeBlue Plus 2 Pre-stained ladder (Thermo Fisher Scientific, myosin: 198 kDa, phosphorylase: 98 kDa, BSA: 62 kDa, glutamic dehydrogenase: 49 kDa, alcohol dehydrogenase: 38 kDa, carbonic anhydrase: 28 kDa, myoglobin red: 17 kDa, lysozyme 14, aprotinin: 6 kDa, and insulin, B chain: 3 kDa). Purified proteins were sequenced by the University of California, San Diego Biomolecular and Proteomics Mass Spectrometry Facility (Table C in S1 File).

Biochemical activity (Table 1) was determined with the model substrate monochlorobimane (Thermo Fisher Scientific) as previously described [22,23,39]. Briefly, reactions were performed in 0.1 M NaCl, 25 mM NaPO_4_, 5% glycerol, pH = 7.0 at 23°C with 50 μM thiol and 50 μM monochlorobimane. The following amounts of protein were used for assays with bacillithiol alone: with the following amounts of protein: BstB: 0.1 μg, BstC: 0.1 μg, BstD: 0.2 μg, BstE: 0.5 μg, BstF: 0.8 μg, BstG: 0.5 μg, BstH: 0.8 μg. Reactions with cysteine, CoA, and bacillithiol + EDTA were performed with 1 mg of each enzyme. Samples were taken every 5, 10, and 20 minutes, and 15 μL samples were quenched in 55 μL 40 mM methanesulfonic acid. HPLC analysis was performed using a 4.6 x 250 mm Beckman Ultrasphere IP C18 column with a linear gradient of 0% of solvent A (0.25% acetic acid, pH = 4.0) to 100% of solvent B (MeOH) over 35 minutes. To determine the metal ion dependence of the enzymes, a solution of enzyme and 1 mM EDTA was incubated in assay buffer for 5 minutes prior to the addition of bacillithiol and monochlorobimane.

**Table 1:** Specific activities of the *Bacillus subtilis* bacillithiol transferases.

*B*. *subtilis* bacillithiol transferase	Specific activity with bacillithiol^a^ (nmol min^-1^ mg^-1^)	Rate of bacillithiol-mB adduct formation (pmol min^-1^)	Rate of cysteine-mB adduct formation (pmol min^-1^)	Rate of Co-mB adduct formation (pmol min^-1^)	Rate of bacillithiol-mB adduct formation + EDTA^b^ (pmol min^-1^)
BstA (YfiT)	2.5 ± 0.4^c^	N/A	2.5 ± 0.2	0.3 ± 0.2	8.7 ± 0.2
BstB (YuaE)	170 ± 13	26 ± 1.1	2.5 ± 0.4	0.3 ± 0.1	8.7 ± 0.1
BstC (YisT)	49 ± 9	14 ± 1.0	2.2 ± 0.1	0.3 ± 0.2	8.9 ± 0.3
BstD (YjoA)	113 ± 6	31 ± 0.8	2.2 ± 0.4	0.3 ± 0.3	8.9 ± 0.2
BstE (YrdA)	5.8 ± 0.7	12 ± 0.9	2.4 ± 0.4	0.3 ± 0.1	8.9 ± 0.2
BstF (YkkA)	1.3 ± 0.2	10 ± 0.4	2.6 ± 0.1	0.3 ± 0.2	8.7 ± 0.1
BstG (DinB)	2.5 ± 0.3	10 ± 0.5	2.2 ± 0.3	0.3 ± 0.3	8.6 ± 0.0
BstH (YizA)	1.8 ± 0.4	10 ± 0.4	2.5 ± 0.4	0.3 ± 0.2	8.5 ± 0.2
No enzyme^d^	-	8.7 ± 0.2	2.6 ± 0.1	0.3 ± 0.3	8.7 ± 0.1

Reactions consisted of 50 μM monochlorobimane and 50 μM thiol, and were conducted at pH = 7.0 at 23°C for a total of 20 min. For reactions with bacillithiol, the following amounts of protein: BstA: 0.1 μg, BstB: 0.1 μg, BstC: 0.1 μg, BstD: 0.2 μg, BstE: 0.5 μg, BstF: 0.8 μg, BstG: 0.5 μg, BstH: 0.8 μg. For reactions with cysteine, CoA, and bacillithiol +EDTA, 1 mg of protein was used. All values represent mean ± standard deviation (n = 3).

^a^Rate of chemical reaction was subtracted from the total rate to give net enzymatic rate.

^b^Enzymes were incubated with 1 mM EDTA in assay buffer for 5 minutes prior to the addition of bacillithiol and monochlorobimane.

^c^Data from Newton *et al* 2011 [22].

^d^“No enzyme” rate is the background chemical reaction rate of 8.7 ± 0.1 pmol min^-1^.

### Minimal inhibitory concentration (MIC) determination and growth curves

Minimum inhibitory concentration (MIC) assays in LB were performed by growing cultures to an OD_600_ of 0.35–0.5 and diluting cells to 5 × 10^7^ CFU/mL in LB. Either LB medium or Spizizen’s minimal medium supplemented with 0.5% casamino acids and 1x trace metals were used in MIC assays, while growth curves were performed in Spizizen’s minimal medium only. For MIC assays in LB medium media, cells were grown in LB to OD_600_ of 0.35–0.5, diluted 1:10, and 10 μL of cells were diluted 1:10 into a 96-well plate containing different concentrations of each antibiotic. For MIC and growth curve assays in minimal medium, cells were grown in LB to OD_600_ of 0.35–0.5, washed in minimal medium twice, and re-suspended to OD_600_ = 1.0, diluted 1:10, and 10 μL of cells were diluted 1:10 into a 96-well plate containing different concentrations of each antibiotic. MIC results were obtained after 24 h incubation at 30°C for LB and 37°C for minimal medium. Plates for growth curves were incubated at 37°C with rotatory shaking and growth was monitored at OD_600_ using an Infinite 200 plate reader from Tecan. Dilutions showing hindered Δ*bshC* mutant growth were selected for further analysis in growth curves. MIC values reported and growth curves shown were calculated from the mean of triplicates (n = 3).

### Fluorescence microscopy data acquisition and analysis

**Fig 2:**
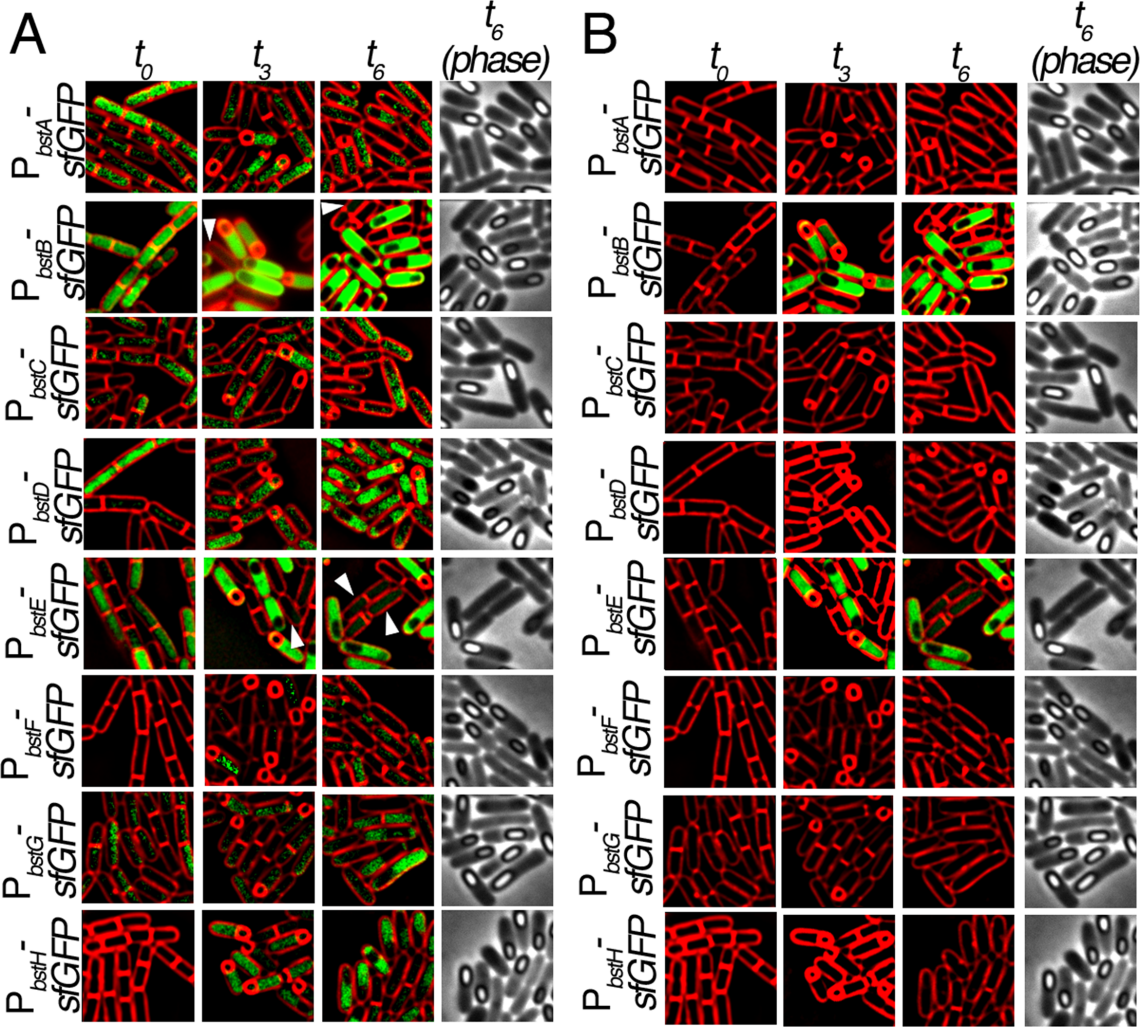
Expression patterns of the *bst* genes during sporulation. Fluorescence microscopy of the eight *bst* promoter fusions to sfGFP during exponential growth (*t*_*0*_), 3 hours (*t*_*3*_) and 6 hours (*t*_*6*_) after induction of sporulation by re-suspension. Cell membranes are stained with FM 4–64 (red). Phase images are shown to visualize cells that are late in the sporulation pathway and contain phase bright forespores. A) Micrographs of the 8 promoters fusions. GFP intensities for each strain and timepoint were adjusted individually to determine which genes were expressed under all conditions. Arrowheads show P_*bstB*_-*sfGFP* and P_*bstE*_-*sfGFP* expression in vegetative cells. B) Micrographs from Fig 2A with the GFP intensity at each panel adjusted to the brightest GFP (P_*bstB*-_*sfGFP* at *t*_6_) to illustrate the relative levels of gene expression. Phase images are shown for *t*_*6*_ images to allow visualization of phase bright intracellular spores at late stages of development.

Samples of sporulating cultures were taken for imaging at the initiation of sporulation (*t*_0_), and 3 hours (*t*_3_), and six hours (*t*_6_) after the induction of sporulation. Eight microliters of cells were added to 2 microliters of a stain mix containing 30 μg ml^−1^ FM 4–64 and 5 μg ml^−1^ DAPI prepared in 1×T-base. Cells were immobilized on an agarose pad (1/10 LB in 1x sporulation medium) and imaged on visualized on an Applied Precision DV Elite optical sectioning microscope equipped with a Photometrics CoolSNAP-HQ^2^ camera. Pictures were deconvolved using SoftWoRx v5.5.1 (Applied Precision) and the medial focal planes are shown. Exposure times for FM 4–64 and GFP were kept constant throughout all experiments. For all images, the phase contrast and FM 4–64 images were adjusted for best visualization. For Fig 2A, the GFP intensity was adjusted for best visualization, and for Fig 2B GFP intensities were normalized between all images to allow for comparisons between strains. To quantify GFP intensity, we manually drew a Region of Interest (ROI) that contains the mother cell and measured the average GFP pixel intensity in ImageJ, and divided by the average GFP pixel intensity of the background (areas in the field that do not contain cells) to calculate fold changes in intensity similar to previous methods[40]. The average of 20 cells is reported here.

### Preparation of lysate for mass spectrometry analysis

Growing cells and sporulating cells were harvested at OD_600_ = 0.4, OD_600_ = 4.0, *t*_*0*_ and *t*_*3*_ of sporulation by centrifugation, suspended in HMS buffer (20 mM HEPES-NaOH, 20 mM MgCl_2_ and 20% sucrose, pH 7.6) containing 100 mM iodoacetamide and treated with 1 mg ml^−1^ lysozyme at 37°C in the dark for 30 min. Spheroplasts were harvested by centrifugation and re-suspended in ice-cold buffer A (20 mM HEPES-NaOH, 150 mM NaCl and 1 mM EDTA, pH 7.6) containing a 1:1000 dilution of protease inhibitor VI, EDTA free (AG Scientific), 10 μg each of DNAse RNAse I, and then treated with 0.5% octylthioglucoside (Thermo Fisher Scientific) on ice for 40 min. The insoluble fraction was removed by centrifugation for 30 minutes at 13,000 RPM at 4°C. Protein amounts were quantified using the Pierce BCA protein assay kit (Thermo Fisher Scientific).

### Mass spectrometry spectral counting

#### Sample preparation and mass spectrometry methods

Lysates were prepared as described above in the presence of alkylating agent iodoacetamide to prevent various reactions of reduced cysteine. Mass spectrometry samples in Table 2 were prepared and analyzed by the University of California, San Diego Biomolecular and Proteomics Mass Spectrometry Facility (http://bpmsf.ucsd.edu/). For in solution digest methods, samples were diluted in TNE (50 mM Tris pH 8.0, 100 mM NaCl, 1 mM EDTA) buffer. RapiGest SF reagent (Waters) was added to the mix to a final concentration of 0.1% and samples were boiled for 5 min. Proteins samples were digested with trypsin (trypsin:protein ratio—1:50) overnight at 37°C. RapiGest was degraded and removed by treating the samples with 250 mM HCl at 37°C for 1 h followed by centrifugation at 14000 rpm for 30 min at 4°C. The soluble fraction was then added to a new tube and the peptides were extracted and desalted using Aspire RP30 desalting columns (Thermo Fisher Scientific). Trypsin-digested peptides were analyzed by HPLC coupled with tandem mass spectroscopy (LC-MS/MS) using nanospray ionization as previously described[41]. Briefly, the nanospray ionization experiments were performed using a TripleTOF 5600 hybrid mass spectrometer (ABSCIEX) interfaced with nano-scale reversed-phase HPLC (Tempo). Peptides were eluted from the C18 column into the mass spectrometer using a linear gradient at a flow rate of 250 μl/min for 3h. MS/MS data were acquired in a data-dependent manner. The collected data were analyzed using MASCOT (Matrix Sciences) and Protein Pilot 4.0 (ABSCIEX) for peptide identifications.

**Table 2:** Adjusted spectral counts of the expression of the bacillithiol transferases and bacillithiol-related proteins during growth and sporulation.

	Adjusted spectral counts^a^			
*B*. *subtilis* protein	Exponential phase	Stationary phase	*t*_*0*_	*t*_*3*_
BstA	ND^b^	ND^b^	ND^b^	ND^b^
BstB	1.5	3.6	10.4	30.2
BstC	ND^b^	ND^b^	ND	ND^b^
BstD	3.8	ND	7.1	4.0
BstE	ND^b^	ND^b^	ND^b^	ND^b^
BstF	ND^b^	ND^b^	ND^b^	ND^b^
BstG	ND^b^	ND^b^	ND^b^	ND^b^
BstH	ND^b^	ND^b^	ND^b^	ND^b^
BrxA	4.5	2.0	5.1	8.1
YtxJ	1.1	12.0	14.9	34.0
CotE^c^	ND^b^	ND^b^	ND^b^	2.8
SpoIIQ^c^	ND^b^	ND^b^	ND^b^	10.6

LC-MS/MS analysis using a TripleTOF 5600 (ABSCIEX) was performed on samples collected during exponential phase (OD600 = 0.4), stationary phase (OD600 = 4.0), at the initiation of sporulation (t0), and 3h after the induction of sporulation (t3). Adjusted spectral counts were calculated by counting the number of peptides with at least 95% confidence that were identified divided by the total amino acid length of the protein.

^a^Total spectral counts for all proteins sum to 1; spectral counts for proteins were multiplied by 10^4^.

^b^ND: not detected.

^c^Sporulation-specific proteins.

#### Spectral counting

Spectral counting was performed as previously described[42]. Spectral counting counts the number of spectra identified peptide in a sample and then integrates the results for all measured peptides of the proteins. For each sample, the adjusted spectral count was calculated for the proteins by counting the number of peptides with at least 95% confidence that were identified, divided by the total amino acid length of the protein. This calculation takes into account that longer proteins generate more peptides and are identified more often because of protein length rather than abundance. Spectral counts were also normalized to the total number of peptides detected in the four growth phases to permit comparisons between samples. The normalized spectral counts were then adjusted by multiplying counts by 10^4^ to allow for ease of visualization.

### Tandem mass tag (TMT) methods and analysis

#### Sample preparation and mass spectrometry methods

Lysate samples in Table 3 were prepared as described above in the presence of alkylating agent iodoacetamide to prevent various reactions of reduced cysteine. Samples were TCA precipitated then re-solubilized in 1M urea and digested with LysC and then trypsin digested. Peptides were desalted using C18 Sep-Paks. Samples were labeled with 10-plex tandem mass tag (TMT) reagents (Thermo Fisher Scientific[43,44]), which are amine-reactive chemical tags, as previously described [45]. This labeling method utilizes tags that are identical in chemical structure, however each tag contains isotopes substituted at various positions resulting in mass reporter and mass normalization regions that have different molecular masses. During MS/MS fragmentation, the reporter ion is released from the labeled peptide and can be used for quantitation. Labeled peptides were fractionated by basic pH reverse-phase liquid chromatography [46] with fraction combining as previously described [47]. Briefly, samples were separated into 96 fractions, pooled into 24 fractions, and 12 fractions were selected for analysis. Fractions were dried and analyzed by LC-MS2/MS3 for identification and quantitation. LC-MS2/MS3 experiments were conducted on an Orbitrap Fusion (Thermo Fisher Scientific) with an in-line Easy-nLC 1000 (Thermo Fisher Scientific).

**Table 3:** Normalized summed signal to noise values of the bacillithiol transferases and bacillithiol-related proteins during growth and sporulation.

	Normalized summed signal to noise values	
*B*. *subtilis* protein	*t*_*0*_	*t*_*3*_
BstA	165.1	388.9
BstB	226.4	192.2
BstC	104.5	243.6
BstD	59.2	36.7
BstE	30.4	97.4
BstF	ND^b^	ND^b^
BstG	180.0	122.2
BstH	ND^b^	ND^b^
BshA	131.0	117.3
BshB1	202.3	186.5
BshB2	196.5	165.5
BshC	338.4	220.0
BrxA	62.6	22.4
BrxB	219.2	113.9
YtxJ	154.9	137.4
YpdA	149.7	154.3
CotE^a^	113.9	334.3
SpoIIQ^a^	9.9	133.5

LC-MS/MS analysis using a Thermo Orbitrap Fusion (Thermo Fisher Scientific) was performed on samples collected at the initiation of sporulation (t0), and 3h after the induction of sporulation (t3). Peptides prepared from PY79 was separated by high pH reverse phase HPLC, fractionated and recombined to produce 10 individual injections of tandem mass tag (TMT, isobaric tag analysis). Normalized summed signal to noise values of the reporter ions were used for quantitation.

^a^Sporulation-specific proteins.

^b^ND: not detected.

#### TMT analysis

Resultant data files were processed using Proteome Discoverer 2.1 (Thermo Fisher Scientific). Reporter ion intensities from TMT reagents were extracted from MS3 spectra for quantitative analysis, and signal to noise values were used for quantitation. See S1 File for details and additional information. Protein level quantitation values were calculated by summing signal to noise values for all peptides per protein meeting the specified filters. Data were normalized and final values are reported as normalized summed signal to noise per protein per sample.

## Results

### Phylogenetic analysis shows the distribution of STL bacillithiol transferase homologs in *Bacillus* species

Previously, we showed that the STL superfamily is extremely divergent, with family members sharing structural rather than sequence similarity[23]. Using the previously described *B*. *subtils* YfiT S-transferase[22] as a query for structural similarity using the Superfamily website[24], we showed that whereas *S*. *aureus* encodes only one STL family member, the bacillithiol transferase BstA, *Bacillus subtilis* encodes a total of eight STL family members, seven of which are putative bacillithiol transferases. Notably the pathogen *Bacillus anthracis* AMES encodes a total of 16 STL family members [22]. To understand the evolutionary relationships between these proteins, we first performed alignments with the primary amino acid sequences of the *B*. *subtilis* enzymes (Figure A in S1 File). This analysis showed that *B*. *subtilis* BstA (formerly YfiT;[23]) and the remaining seven putative *B*. *subtilis* bacillithiol transferases share very low sequence identity: BstA showed between 9–23% identity to the remaining transferases, and the most closely related pair (YisT and DinB) showed just 37% sequence identity. Thus, *B*. *subtilis* 168 encodes eight STL family members distantly related to one another.

To further understand the diversity of these enzymes and their distribution across other *B*. *subtilis* strains, we next constructed a phylogenetic tree that contains STL superfamily members from *B*. *subtilis* strain 168 and four other *B*. *subtilis* strains. Due to its lack of sequence or structural similarities to the STL superfamily bacillithiol transferases, *B*. *subtilis* FosB bacillithiol transferase served as outgroup for this analysis (black, Fig 1). BstG/DinB and BstC/YisT are more closely related to each other than to any other putative bacillithiol transferase and therefore cluster closely together on a phylogenetic tree (green, Fig 1). We found that seven STL homologs are conserved in all five strains, shown as proteins that branch and cluster together (Fig 1A), and share ≥88% sequence similarity with the *B*. *subtilis* 168 protein sequences (Table A in S1 File). The eighth branch (defined by BstH/YizA) is found in *B*. *subtilis* 168 as well as the two *B*. *subtilis spizizenii* strains analyzed. Interestingly, *B*. *subtilis subsp*. *spizizenii* TU-B-10 encodes an extra STL enzyme (AEP87498.1) that even its closest relative, *B*. *subtilis subsp*. *spizizenii* W23, does not encode. This protein, AEP87498.1, does not cluster with any of the STL enzymes encoded in the five *B*. *subtilis* strains (Fig 1A). In addition, a BLAST search shows that the same protein (100% sequence identity and coverage) is found in *Jeotgalibacillus marinus*. Thus, the *B*. *subtilis* strains we examined encode seven of the eight STL-related proteins found in the *B*. *subtilis* 168 genome, and two of these proteins (BstH/YizA from 168, and AEP87498.1 from TU-B-10) show a more limited distribution, perhaps indicating that they perform a specialized function. This demonstrates that even within a single species, individual strains show a degree of variability in their repertoire of STL-family members.

To study the conservation of STL proteins different *Bacillus* species, we performed a phylogenetic analysis on the proteins encoded by *B*. *subtilis* 168, *Bacillus megaterium* QMB1551, *Bacillus amyloliquefaciens subsp*. *plantarum str*. FZB42, *Bacillus halodurans* C-125, *Bacillus anthracis str*. *Ames*, and *Bacillus thuringiensis* str. Al Hakam (Fig 1B). We find that most of the STL proteins are found in all six species analyzed, with some species even encoding more than one copy of the same STL homolog with ≥30% sequence identity, while some homologs are missing (Fig 1B, Table B in S1 File). For example, a *B*. *subtilis* BstC/YisT homolog is not present in *Bacillus anthracis Ames*. Additionally, there are a number of homologs that are encoded in other species but are not in *B*. *subtilis* 168. For example *B*. *anthracis* Ames and *B*. *thuringiensis* str. Al Hakam encode eight and eleven STL homologs, respectively, not encoded in *B*. *subtilis*. Thus, there is substantial variability in the STL superfamily of proteins between related species, indicating that there is evolutionary pressure to retain and diversify the STL superfamily of enzymes.

The diversity of the STL-superfamily members encoded in different strains and species suggests that these enzymes might be part of the dispensable genome of *Bacillus* species, rather than the core genome. Indeed, a recent genomic study of the genomes of 20 *Bacillus* species identified 814 conserved genes that comprise the core genome of *Bacillus* species[48]. This study revealed that the bacillithiol biosynthesis genes *bshA*, *bshB2*, and *bshC*, but none of the eight *B*. *subtilis* bacillithiol transferases, are part of the core genome[48]. However, we note that each of the specific strains included in this study encode STL-family proteins, suggesting that the function may be required. Indeed, the published study included *B*. *coahuilensis* (NZ_ABFU00000000), which has the smallest genome reported for a *Bacillus* species. BLAST searches revealed that the *B*. *coahuilensis* genome encoded homologs of just two of the eight *B*. *subtilis* proteins, BstA (for which WP_010173769.1 showed 97% coverage and 51% sequence identity) and BstB/YuaE (for which WP_010173272.1 showed 85% coverage and 32% sequence identity). BstA and/or BstB/YuaE homologs were found in all the species we analyzed, except *Bacillus clausii* KSM-K16 (NC_006582) and *Oceanobacillus iheyensis* HTE831 (NC_004193), which encoded other members of the STL superfamily that were identified by the PFAM analysis. Thus, all of the genomes we analyzed encode at least one putative bacillithiol transferase, suggesting that this activity is conserved, although none of the *B*. *subtilis* enzymes are conserved across all twenty species.

### Biochemical activity of the putative bacillithiol transferases

In order to test if all the STL-related proteins encoded by *B*. *subtilis* strain 168 were active bacillithiol transferases, we overexpressed and purified the His_6_-tagged proteins (Figure B in S1 File and “Materials and Methods”). We assessed the specific activities of the purified putative bacillithiol transferases using the electrophile monochlorobimane. Monochlorobimane reacts slowly with thiols, and upon addition of a bacillithiol transferase, the rate of product formation increases[23]. Accumulation of the product (bacillithiol-bimane) can be monitored over time using reverse phase HPLC and fluorescence detection. This analysis showed that all seven enzymes were active to varying degrees with bacillithiol and monochlorobimane, with all showing specific activities that are above the chemical rate background (Table 1). We therefore renamed the proteins BstB-H, replacing their provisional ORF names with *bstB* (*yuaE*), *bstC* (*yisT*), *bstD* (*yjoA*), *bstE* (*yrdA*), *bstF* (*ykkA*), *bstG* (*dinB*), *bstH* (*yizA*). Four of these enzymes (BstB, BstC, BstD, and BstE) have specific activities significantly higher than *B*. *subtilis* BstA[22]. These high activity enzymes (Table 1) have rates that compare well with those found for bacterial glutathione transferases (160–5000 nmole min^-1^mg^-1^[49]). After subtracting the chemical rate (8.7 ± 0.1 pmol min^-1^), the specific activity of BstG is comparable to the specific activity reported for *B*. *subtilis* BstA, while that of BstF and BstH are lower than that of BstA[22]. The low activity of these enzymes could be due either to the His_6_-tags, which could adversely affect enzyme activity of these purported metalloenzymes, or it could be due to a poor fit between the active site and monochlorobimame, which is unlikely to be the natural substrate for any of these enzymes.

We next tested the specificity of these enzymes for its co-substrate bacillithiol versus two other major low molecular weight thiols found in *B*. *subtilis* cells, cysteine and coenzyme A (CoA). The bacillithiol transferases were unable to catalyze formation of cysteine-bimane or CoA-bimane above the background chemical rates (Table 1). Thus, the *B*. *subtilis* bacillithiol transferases are inactive with either cysteine or CoA. Together, these data indicate that despite the low sequence similarity to each other or to *S*. *aureus* BstA, all eight of the putative bacillithiol transferases encoded by the *B*. *subtilis* 168 genome are active and that they use bacillithiol as a co-substrate. These results demonstrate that widely divergent members of the STL-superfamily retain a common enzymatic activity.

### BstA-H activity is metal dependent

The crystal structure of *B*. *subtilis* BstA has been shown to have an active site geometry consistent with those of metalloenzymes, with three conserved histidines (H67, H160, and H164) that might coordinate an active site metal[50]. The authors of this study therefore predicted that the proteins might be metal dependent, and indeed, we previously determined that the activity of bacillithiol transferase from *S*. *aureus* is metal dependent [23]. An alignment of the eight bacillithiol transferases of *B*. *subtilis* 168 (Figure A in S1 File) revealed that these three histidines were the only completely conserved amino acids. The His-tagged bacillithiol transferases were presumed to be in the zinc form due to the use of zinc affinity columns during purification. To determine the metal ion requirement of the eight purified bacillithiol transferases, we added the divalent cation-chelating agent EDTA to the monochlorobimane enzyme assay to establish whether addition of this chelating agent would decrease enzyme activity. Upon addition of EDTA, the activity of all eight enzymes was decreased to a rate comparable to the chemical rate, which represents a >99% reduction in rate (Table 1). These data suggest that the activity of all eight bacillithiol transferases is metal-dependent, although further studies are required to determine which metal is normally used by the enzymes *in vivo*. Such metalation studies of thiol transferases have revealed the substrate specificity and enzymatic rate may vary with metal ion[21,51].

### Phenotypic analysis of the Δ*bshC* and Δ*bstA-H* mutants against select oxidants and stressors

Bacillithiol is implicated in a number of protective as well as physiological roles. It has been found to detoxify fosfomycin [15,21], to serve as a buffer for cytoplasmic zinc [52], to protect cysteine residues from oxidative reactions during NaOCl stress [16,17], and to contribute to the assembly of Fe(S) clusters [18] in *B*. *subtilis*. Bacillithiol-deficient mutants of *B*. *subtilis* have been reported to grow slowly in minimal media and to be sensitive to NaOCl, paraquat, CdCl_2_, and CuCl, which all cause oxidative stress in minimal media [18] and to high concentrations of NaCl[15]. It is possible that bacillithiol-dependent protective reactions are catalyzed by bacillithiol transferases, so we tested the sensitivity of *bst* mutants to fosfomycin, paraquat, CdCl_2_, CuCl, and NaCl in both nutrient-rich LB and minimal media. We first constructed in frame deletions of all eight *bst* genes (as described in Materials and Methods). To account for potential functional redundancy among the proteins, we then combined these eight mutations into a single strain, generating a strain containing Δ*bstA*, Δ*bstB*, Δ*bstC*, Δ*bstD*, Δ*bstE*, Δ*bstF*, Δ*bstG* and Δ*bstH* (hereafter referred to as Δ*bstA-H*). This strain was compared to the bacillithiol deficient Δ*bshC* strain and the wild type strain PY79. The minimum inhibitory concentration (MIC) was determined 24 hours after inoculation with the stressor. As expected, the Δ*bshC*, but not the Δ*bstA-H* mutant, was more sensitive to fosfomycin, which is detoxified by the FosB transferase, than PY79 in both minimal and rich media. However, the wild type PY79, the Δ*bshC* and Δ*bstA-H* strains shared the same MIC in both types of media against all of the other stressors (Table D in S1 File), suggesting that these strains were not sensitized to any of the stressors. However, the MIC is an endpoint assay that could miss decrease in growth rate and longer lag phases, so we also performed growth curves for all three strains in the presence of stressors. The Δ*bstA-H* strain showed growth that was identical to the wild type strain under all conditions, demonstrating that the proteins are not involved in resistance to these agents. The Δ*bshC* mutant showed a slightly increased lag in growth with CdCl_2_, NaOCl and paraquat (Figure C in S1 File), although the growth defects we observed were less severe than previously reported[16,53]. Thus, the strain lacking all eight of the bacillithiol transferases identified so far shows normal growth in the presence of a variety of stressors.

### Mass spectrometry analysis and fluorescence microscopy show that bacillithiol transferases and bacilliredoxins are expressed during growth and sporulation

We reasoned that studying the regulation of the bacillithiol transferases might help reveal their endogenous functions. For example, the four genes required for bacillithiol biosynthesis, *bshA*, *bshB1*, *bshB2*, and *bshC*, are under the control of the Spx transcription factor[54], which is activated by stressors such as oxidants, electrophiles, and heat. In addition, *bshA* and *bshB1* have been reported to be under the control of the sporulation-specific transcription factor sigma E, which controls early mother cell-specific transcription[55]. However, there is very little documentation in the literature about the expression of the bacillithiol transferases. We first used mass spectrometry analysis to examine the expression of bacillithiol transferase during different stages of growth. To do so, we used spectral counting of native peptides from a *B*. *subtilis* PY79 extract to assess the total number of spectra identified for a peptide to quantify the relative abundance of each bacillithiol transferase[42,56,57]. Using a single injection of extract separated using a low pH capillary HPLC separation online with QTOF mass spectrometer we were able to detect a total of 762 proteins, with 390, 238, 521 and 690 proteins (S1 Dataset) detected in exponential phase, stationary phase, *t*_*0*_ and *t*_*3*_ of sporulation, respectively. We detected BstB expression during exponential phase (OD_600_ = 0.4), stationary phase (OD_600_ = 4.0), *t*_*0*_, and *t*_*3*_ of sporulation, while BstD expression was detected during exponential phase, *t*_*0*_, and *t*_*3*_ of sporulation (Table 2). BstD was expressed constitutively whereas BstB appeared to increase 2.9-fold between *t*_*0*_ and *t*_*3*_ of sporulation. Interestingly, the confirmed bacilliredoxin BrxA [58] and the putative bacilliredoxin YtxJ[15] are also expressed during growth and sporulation, with YtxJ increasing 2.3-fold during sporulation, following a pattern similar to that of BstB (Table 2).

It is possible that we failed to detect other bacillithiol transferases because high abundance peptides masked those from lower abundance proteins of interest in these complex extracts. To remedy this and allow for increased protein and peptide identification, we used a high-pH reversed-phase fractionation method where single samples were separated offline into 96 fractions, pooled, dried, and injected at low pH as 12 individual samples as previously described[46,59]. These methods complemented the low pH, single injection method used in Table 2. We also utilized a more sensitive instrument, a Thermo Orbitrap Fusion, to gain greater coverage of the *B*. *subtilis* proteome [60,61]. For quantitation purposes, samples were labeled with tandem mass tag (TMT) reagents as previously described[45]. We chose to examine cells at the initiation of sporulation (*t*_*0*_), and three hours after the initiation of sporulation (*t*_*3*_) because this would give us information about stationary phase as well as sporulation. Indeed, using these methods we identified 2837 proteins (S2 Dataset), a large proportion of the 4100 theoretical *B*. *subtilis* proteins, as compared to the 762 proteins identified in Table 2. We detected all of the bacillithiol transferases except for BstF and BstH during both time points (Table 3). We also detected all four bacillithiol biosynthesis genes, the three confirmed and putative bacilliredoxins, and the putative bacilliredoxin reductase YpdA. Notably, BstA and BstE expression increased almost 3-fold during sporulation.

We further examined expression of the bacillithiol transferases by using GFP reporter constructs to determine if the proteins were expressed uniformly in cells within the population and to see if we could detect expression of BstF and BstH. Our mass spectrometry analysis suggested that some of these proteins were induced during sporulation, and we were therefore also interested in determining if they were expressed in the vegetative or the sporulating cells in these cultures, and if the latter, if induction was occurring in a cell specific manner. We therefore constructed strains containing fusions of the promoters for each of the eight bacillithiol transferase genes (*bstA-H*), to superfolder green fluorescent protein (sfGFP) and monitored the cells during growth and sporulation using fluorescence microscopy. We were able to detect expression of all of the *bst* genes except *bstF* and *bstH* immediately after the initiation of sporulation by resuspension (*t*_*0*_, Fig 2A) at levels similar to that seen in stationary phase cells. The *bstB* and *bstE* genes were expressed at the highest levels, and most cells showed similar levels of expression. The other genes were expressed at lower levels, with substantial cell-to-cell variability. Many *bst* genes were also expressed during sporulation, and several appeared to be substantially upregulated in the larger mother cell during sporulation, including *bstB*, *bstD*, *bstE*, and *bstF*. In some of these strains, low levels of GFP expression were also seen in some vegetative cells (Fig 2A *bstB* and *bstE*, arrowheads). Some genes, such as *bstA*, *bstC*, *bstG*, and *bstH*, were expressed in both vegetative and sporulating cells, in most cases with substantial heterogeneity in expression levels in both cell types.

To more readily compare GFP intensity and gene expression across strains and time points, the GFP intensity was adjusted in an identical manner relative to the brightest strain and timepoint (*bstB* at *t*_6_) for images collected with the same exposure time (Fig 2B). This indicated that of the eight genes, *bstB* and *bstE* showed the highest expression, and that they were induced in the mother cells of sporangia at *t*_3_ and *t*_6_ of sporulation. Using these adjustment methods, GFP from the other genes could not be detected. Quantification of *bstB* and *bstE* expression at *t*_*3*_ (as described in Materials and Methods) revealed that expression of P_*bstB*_*-GFP* and P_*bstE*_*-GFP* was 30-50x higher in the mother cell than background. Similar results were obtained with BstB-GFP and BstE-GFP protein fusions. Thus, of the eight *bst* genes, low and often heterogeneous levels of expression was detected in vegetative cells and in the mother cell of sporulating cells for several proteins, with high mother cell specific expression observed for the two most highly expressed *bst* genes, *bstB* and *bstE*. Together, our mass spectrometry and microscopy data show that all of the bacillithiol transferases are produced to variable levels under at least one condition during growth and sporulation, although most are expressed at substantially lower levels than *bstB* and *bstE*.

### Bacillithiol, bacillithiol transferases, and bacilliredoxins are not essential for sporulation

We next tested if bacillithiol and the bacillithiol transferases are required for sporulation, as suggested by the above results and as reported by a previous study, which reported that the *bshA* deletion of *B*. *subtilis* CU1065 shows a ~100-fold reduction in spore titer [15]. To test this hypothesis, spore titers were determined by enumerating the number of heat resistant colony forming units per mL after 24 hours growth in DSM for PY79, derivatives with the Δ*bshA*, Δ*bshC*, Δ*bstA-H*, and Δ*spo0A* mutations and the Δ*bshA* mutant in a CU1065 background. We also constructed a strain deficient in the confirmed bacilliredoxins *brxA* and *brxB* and the putative bacilliredoxin *ytxJ*, hereafter referred to as Δ*brxA*Δ*brxB*Δ*ytxJ*, to assess the role of bacilliredoxins in spore formation and germination. The Δ*bshA* and Δ*bshC* mutants were included to assess the role of bacillithiol in sporulation. We included two sporulation defective control strains, Δ*spo0A* which is defective in biofilm formation and early sporulation specific gene expression, and Δ*sigH*, an alternative sigma factor that directs the transcription of genes that function during the transition from exponential growth to stationary phase.

We found no difference in the spore titer in *B*. *subtilis* PY79, and strains with Δ*bshA*, Δ*bshC*, Δ*bstA-H*, or Δ*brxA*Δ*brxB*Δ*ytxJ* mutations: each produced similar number of heat resistant spores (Table 4). We therefore tested the impact of the *bstA* mutation in *B*. *subtilis* strain CU1065, and observed a ~14-fold reduction in spore titer in the Δ*bshA* strain, smaller than the previously observed ~100-fold reduction previously reported, which might be attributed to differences in the media, or assay method used [15].

**Table 4:** Spore titers of relevant strains.

Strain	Relevant genotype	Spore titer (cfu mL^-1^)^a^
CU1065	Parental strain	3.8 x 10^8^
HB11002	Δ*bshA*	2.6 x 10^7^
PY79	Parental strain	1.6 x 10^9^
KP648	Δ*spo0A*	≤10^2^
BER657	Δ*sigH*	≤10^2^
AD3303	Δ*bshA*	1.7 x 10^9^
RP82	Δ*bshC*	1.3 x 10^9^
RP238	Δ*bstA-H*	1.2 x 10^9^
RP259	Δ*brxA*, Δ*brxB*, Δ*ytxJ*	1.9 x 10^9^

^a^Titers were determined as described in the “Materials and Methods.” The values shown are averages of at least two experiments.

Differences in the spore titers of the bacillithiol-deficient mutants can be attributed to differences between the PY79 and CU1065 parental strains utilized. We also used microscopy to monitor the timing of engulfment [62] and germination [63] for the *B*. *subtilis* PY79 Δ*bshA*, Δ*bshC* and Δ*bstA-H* strains, and both processes appeared to occur normally. To test for more subtle defects in spore assembly or function, we tested the Δ*bshC* and Δ*bstA-H* spores for resistance to oxidants, lysozyme, and ethanol, finding them to be as resistant as wild type (Table E in S1 File). Thus, in our studies we were unable to detect any change in the durability of the mutant Δ*bshC* and Δ*bstA-H* spores, indicating that the integrity of the spore coat and the dehydration of the spore appear to remain uncompromised in the absence of bacillithiol and all eight identified bacillithiol transferases in the STL superfamily. Thus, it remains unclear what function these proteins serve in sporulating cells.

## Discussion

The STL superfamily of thiol transferases is a large, divergent family of enzymes that are related by structural rather than sequence similarity. Most of the STL bacillithiol transferases encoded in *Bacillus subtilis* share less than 30% sequence similarity and would not be considered to be part of the same protein family. Yet, we here demonstrate that each of the predicted STL enzymes[22] is active to varying degrees with the model substrate monochlorobimane. Our data indicates that like *S*. *aureus* BstA[23], these enzymes are active with bacillithiol, but not with cysteine or Coenzyme A, and that they are metalloenzymes. The lowest activity was observed for BstF, BstG and BstH, while BstB and BstD showed a very high enzymatic activity under the conditions used here. We have therefore renamed the enzymes according to the STL nomenclature used previously (*bstA-H* [23])in order to avoid confusion with the old DinB/YfiT-like nomenclature. It is possible that the lower activity of some of these enzymes is due to the use of the incorrect metal cofactor, inclusion of the His_6_ tag [64], or a low binding affinity between the active site and the model substrate monochlorobimane.

Phylogenetic analysis shows that none of the eight bacillithiol transferases encoded by *B*. *subtilis* 168 are part of the core genome [48], although seven are found in all four *B*. *subtilis* strains that we here investigated in more detail. Two strains do not encode BstH/YizA, while *Bacillus subtilis subsp*. *spizizenii* TU-B-10 which is an environmental strain isolated from Nefta, Tunisia[65], encodes an additional bacillithiol transferase that is not encoded in the other *B*. *subtilis* strains. Interestingly, an identical protein is found in *Jeotgalibacillus marinus*, which is a marine strain that is found in the traditional Korean food jeotgal[66,67]. When the phylogenetic analysis is expanded to include other *Bacillus* species, we find an even greater diversity of STL enzymes, with different species encoding additional homologs. Like *B*. *subtilis*, *B*. *amyloliquefaciens* encodes a total of eight STL enzymes, but one enzyme does not share homology with any of the *B*. *subtilis* STL bacillithiol transferases. *B*. *halodurans*, *B*. *anthracis*, *B*. *thuringiensis*, and *B*. *megaterium* encode four, eight, eleven, and six additional STL enzymes, respectively. Thus, different strains and species show variability in their suite of putative bacillitiol transferases.

Currently the natural substrates of any of the *B*. *subtilis* bacillithiol transferases are unknown. However, recent studies show two mycothiol transferases in the STL family are involved in the biosynthesis [13], rather than the detoxification, of secondary metabolites, which is a new and interesting role for thiol transferases. The STL superfamily member LmbV transfers mycothiol to a precursor molecule during lincomycin A biosynthesis in *Streptomyces lincolnensis*, incorporating the sulfur of mycothiol into the final Lincomycin A molecule [13]. Additionally, the STL superfamily enzyme EgtB was determined to catalyze the transfer of γ-glutamyl cysteine to N-α-trimethyl histidine during ergothioneine biosynthesis in *Mycobacterium thermoresistibile*[14]. In both cases, the authors note that the STL mycothiol transferases contain two Pfam domains: the first being the DinB domain responsible for thiol transfer, the second being a domain with a different function. This second domain most likely drives the specificity toward the non-thiol co-substrate. Characterization of the second domain could aid in the quest to find the natural substrates of multi-domain thiol transferses. In the case of the eight *B*. *subtilis* STL bacillithiol transferases, however, each enzyme contains only a single domain, the DinB domain. Thus, a different strategy must be used to identify the natural substrates of these enzymes.

We took two different approaches to begin the task of identifying the physiological roles and natural substrates of the bacillithiol transferases. First, we took a candidate approach in which we tested the sensitivity of the Δ*bstA-H* mutant lacking all members of the STL family to molecules that bacillithiol-deficient strains are sensitive to in nutrient rich and nutrient limiting media. We assessed sensitivity in MIC format, where growth is assessed 24 hours after inoculation, and in growth curves, where growth is monitored from the time of inoculation through stationary phase. In both cases we found no molecules that Δ*bstA-H* is sensitive to and in fact, determined that growth of the Δ*bshC* mutant was not as severely reduced by stressors as previously reported [16,53], with the sole exception of fosfomycin. The reasons for this discrepancy might be due to the different strains used or differences in the levels of trace metals or other components in the media. Thus, we were unable to find a growth defect associated with the absence of the bacillithiol transferases, in the presence or absence of various stressors.

In the second approach, we monitored *bst* gene expression during different phases of *B*. *subtilis* growth and development, hypothesizing that the enzymes might be involved in either morphogenesis or in resistance to endogenous toxins and oxidants. We used GFP fusions and two complementary mass spectrometry methods to assess expression of bacillithiol transferases during vegetative growth and sporulation. Using mass spectrometry, we were able to demonstrate expression of all bacillithiol transferases except BstF and BstH, as well as the bacillithiol biosynthesis proteins, and the confirmed and putative bacilliredoxins during growth and sporulation. We also used GFP reporter strains to visualize the spatial and temporal expression of *bst* promoter fusions to GFP during growth and sporulation. Using this method, we determined that six of the *bst* genes are expressed at variable and low levels at during growth, and that all eight *bst* genes are expressed during sporulation. Several of the promoters are induced in the mother cell, with very high mother cell specific expression of *bstB* and *bstE*. However, we found that mutants lacking bacillithiol or all eight bacillithiol transferases have no impact on sporulation in PY79, although we found a ~14 fold reduction in spore titer of bacillithiol-deficient mutants in one *B*. *subitlis* strain. Further studies are necessary to determine the role that the bacillithiol transferases might play in sporulation and to understand the strain specificity of the impact. It is possible that bacillithiol transferases contribute to mother cell specific processes, such as assembly or modification of the proteinaceous spore coat, or that they protect the developing spore from exogenous toxins and oxidants.

**Fig 3:**
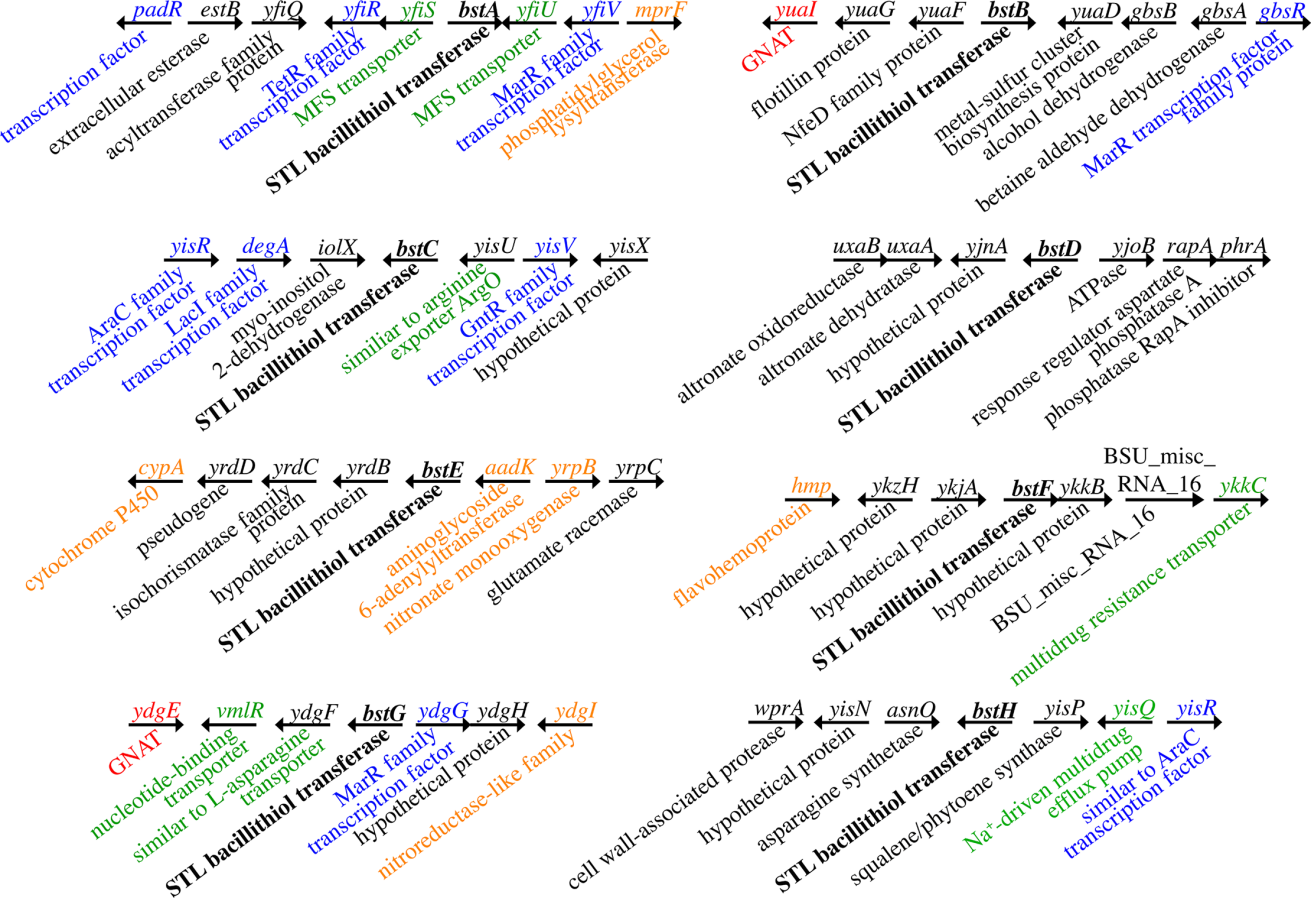
The genomic contexts of the eight STL bacillithiol transferases. Gene symbols are listed above the arrow and gene descriptions are listed below the arrow. Descriptions are annotated from NCBI and SubtiList. STL bacillithiol transferase genes are bolded. Green: transporters, blue: transcription factors, red: GNAT family members, orange: drug and oxidative stress resistance related genes.

The genomic context of the genes encoding the bacillithiol transferases may also provide information about the physiological roles and the natural substrates for these enzymes. Many of the genes in the vicinity of the bacillithiol transferases are involved in efflux and transcription, suggesting a possible role for some enzymes in detoxification of specific molecules (Fig 3). Prior studies have demonstrated that after bacillithiol is conjugated to the target substrate, a bacillithiol conjugate amidase hydrolyzes the bacillithiol, and that an *N*-acetyltransferase in the GNAT family transfers acetyl to the resulting cysteine to form a mercapturic acid adduct, which exits the cell via passive diffusion or transport[22,53,68–70]. BshB2 has been shown to have bacillithiol conjugate amidase activity in *B*. *anthracis* and *S*. *aureus* [23,53,70], but the *N*-acetyltransferases and efflux pumps have yet to be identified. Interestingly, several genes encoding proteins with sequence similarity to the GNAT family of *N*-acetyltransferases and efflux transporters are found in close proximity to *bst* genes, including *yisL* and *vlmR*, which are found next to each other. Other interesting genes in the vicinity include a wide array of transcription factors that might provide insight into bacillithiol expression and *yisP*, which encodes a farnesyl diphosphate phosphatase involved in the synthesis of squalene. Farnesol, the product of YisP contributes to biofilm formation in *B*. *subtilis* and modifies lipid bilayer structure to convey oxidative resistance much like the *S*. *aureus* virulence factor staphyloxanthin[71]. Staphyloxanthin biosynthetic genes are upregulated during thiol stress in a bacillithiol deficient mutant of *S*. *aureus*, although staphyloxanthin levels are the same in the different strains[72]. Thus, it would be interesting to see if bacillithiol affects biofilm formation and membrane integrity in *Bacillus subtilis*.

Our studies have confirmed the activity of a new superfamily of bacillithiol transferases encoded in *Bacillus subtilis*. We show that these enzymes are extremely divergent in sequence and display a wide range of biochemical activity. The high level of divergence among the *B*. *subtilis* bacillithiol transferases suggests that they are under selective pressure, perhaps to cope with an equally diverse array of endogenous and/or exogenous toxic metabolites and oxidants. Indeed, additional studies of the enzymes are needed to determine the physiological roles of these enzymes. Given the data presented here, we believe stationary phase and sporulation might be appropriate places to start.

## Supporting information

S1 File Supporting information file. Figures and tables containing supporting data.
(DOCX)

S1 Dataset Mass spectrometry data. Spectral counts for Table 2.
(XLSB)

S2 Dataset Mass spectrometry data. Normalized summed signal to noise for Table 3.
(XLSX)
