# Supplementary material for: Identification of the S-transferase like superfamily bacillithiol transferases encoded by Bacillus subtilis
Source: PLoS One. 2018 Feb 16;13(2):e0192977. doi: 10.1371/journal.pone.0192977 (PMC5815605; doi:10.1371/journal.pone.0192977)
Supplement: S1 File — Figures and tables containing supporting data. (DOCX) [file pone.0192977.s001.docx]

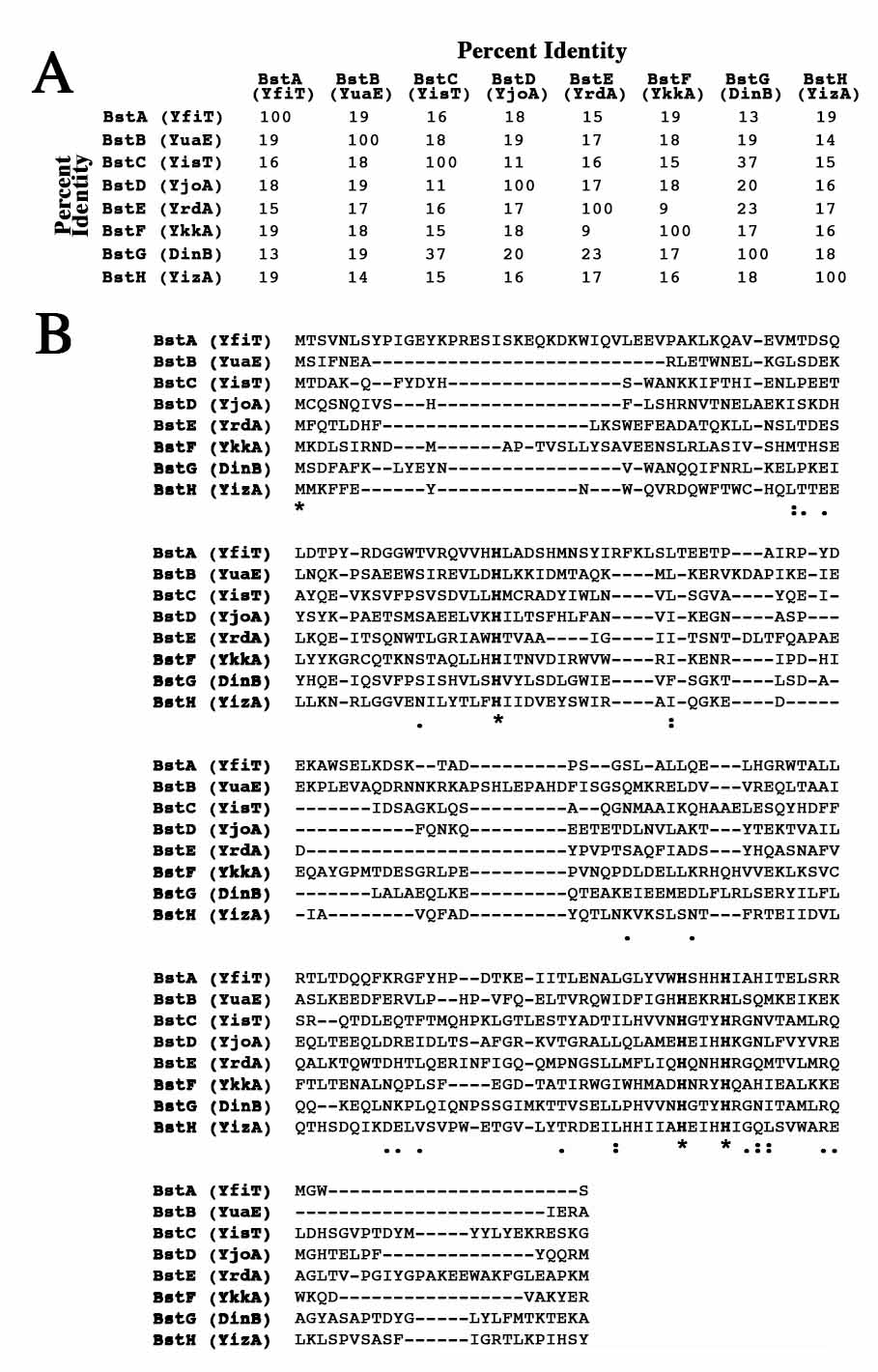


**Figure A**: **Bioinformatic analysis of the eight *B. subtilis* bacillithiol transferases.** A) Percent sequence identity of the bacillithiol transferases B) Full sequence alignments of the bacillithiol transferases.


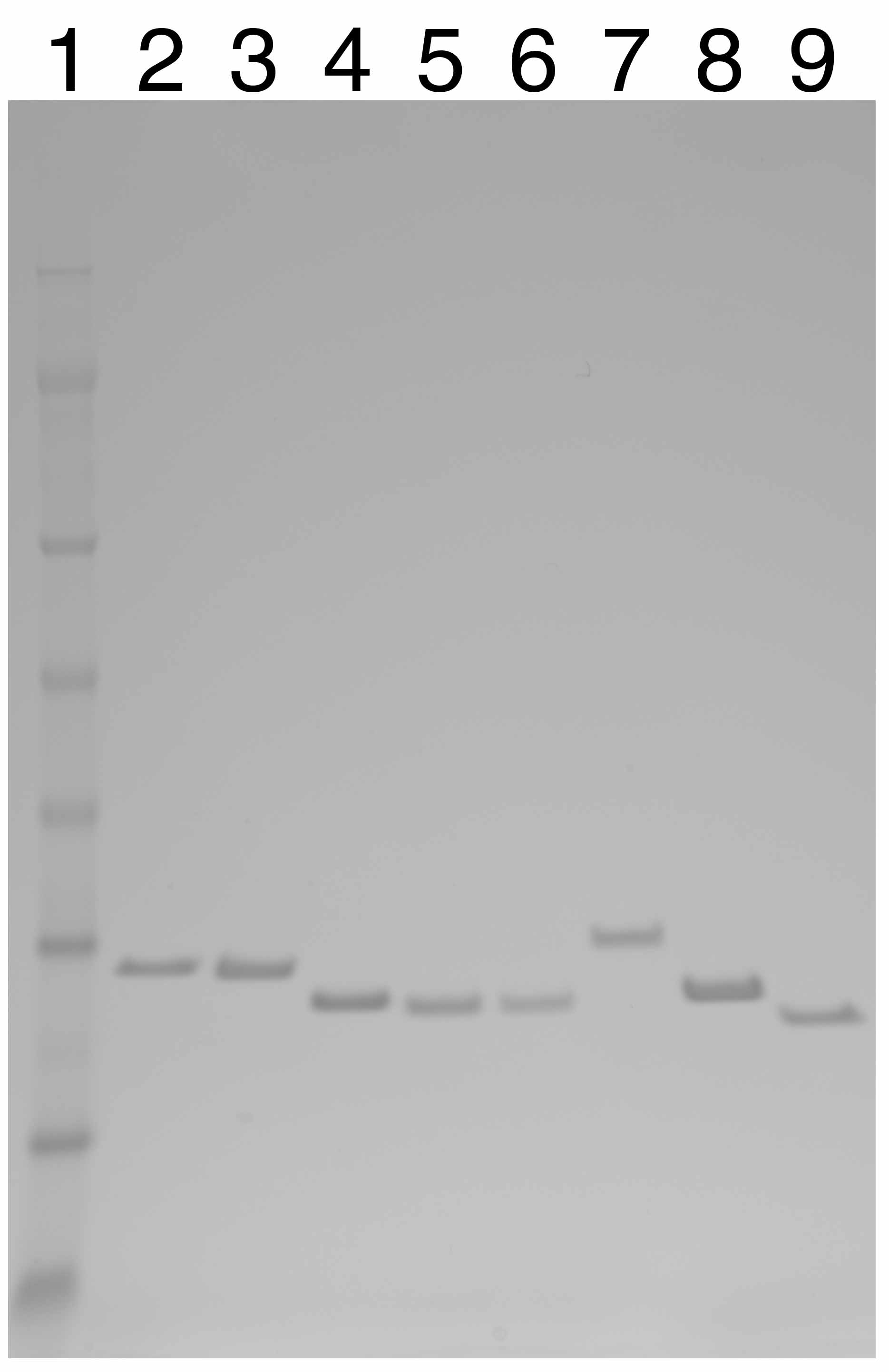


**Figure B: SDS PAGE analysis of the purified His_6_-tagged *B. subtilis* STL bacillithiol transferases.** Analysis of recombinant His_6_-tagged BST enzyme purification on a reducing 4-12% Bis-Tris SDS-PAGE gel. Lane 1, ladder; lane 2, BstA/YfiT (purified protein published previously[[1](#_ENREF_1)]); lane 3, BstB/YuaE; lane 4, BstC/YisT; lane 5, BstD (YjoA); lane 6, BstE (YrdA); lane 7, BstF (YkkA); lane 8, BstG (DinB); lane 9, BstH (YizA).

**
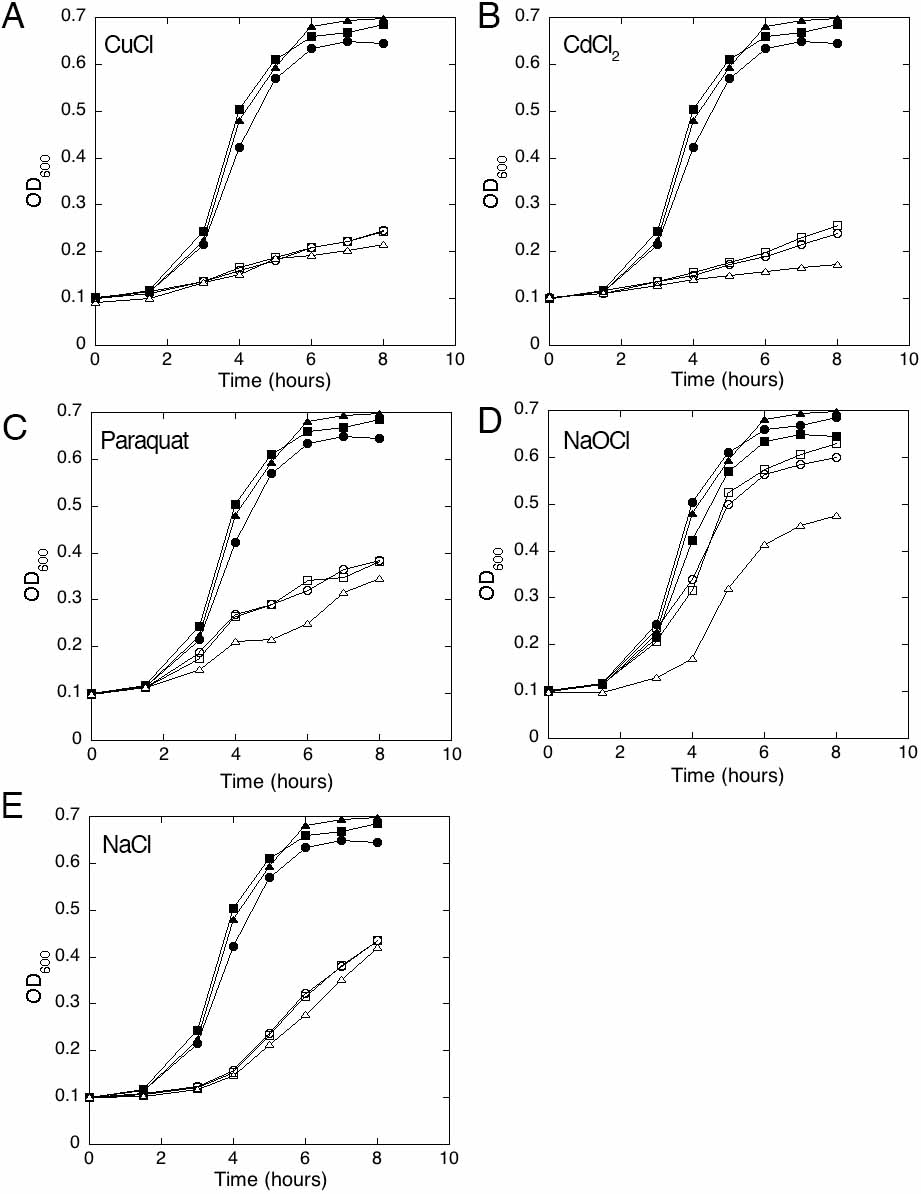
**

**Figure C: Growth curves of wild type *B. subtilis* PY79, Δ*bstA-H* mutant, and Δ*bshC* mutant in the presence of stressors.** Cultures were grown in Spizizen’s minimal media supplemented with 0.5% casamino acids and 1x trace metals solution (see Materials and Methods). The cell growth rate was monitored at OD_600_ using an Infinite 200 plate reader from Tecan. Filled symbols: untreated; open symbols: treated. Circles: PY79, squares: Δ*bstA-H* mutant, triangles: Δ*bshC* mutant. The curves shown are calculated from the mean of three experiments. A) CuCl treated cells 4x below the MIC (0.6 mM) B) CdCl_2_ treated cells 4x below the MIC (6.25 μM) C) Paraquat treated cells 4x below the MIC (6.25 μM) D) NaOCl treated cells 4x below the MIC (125 μM) E) NaCl treated cells 2x below the MIC (625 mM).

| **Table A: Bacillithiol transferase homologs in different *Bacillus subtilis* strains** | | | | |
| --- | --- | --- | --- | --- |
| ***Bacillus subtilis 168*** | ***Bacillus subtilis subsp. natto* BEST195**  (% sequence ID, % sequence coverage) | ***Bacillus subtilis subsp. subtilis***  **str. RO-NN-1**  (% sequence ID, % sequence coverage) | ***Bacillus subtilis subsp. spizizenii***  **str. W23**  (% sequence ID, % sequence coverage) | ***Bacillus subtilis subsp. spizizenii***  **TU-B-10**  (% sequence ID, % sequence coverage) |
| BstA (YfiT) | BAI84358.1 (98,100) | AEP89936.1 (97, 100) | ADM36901.1 (93, 100) | AEP85784.1 (93, 98) |
| BstB (YuaE) | BAI86618.2 (99, 100) | AEP92104.1 (100, 100) | ADM39050.1 (93, 100) | AEP87960.1 (96, 100) |
| BstC (YisT) | BAI84646.1 (98, 100) | AEP90192.1 (95, 100) | ADM37154.1 (86, 100) | AEP86047.1 (88, 100) |
| BstD (YjoA) | BAI84830.2 (99, 100) | AEP90365.1 (97, 100) | ADM37313.1 (95, 100) | AEP86196.1 (97, 100) |
| BstE (YrdA) | BAI86152.1 (98,100) | AEP91657.1 (98, 100) | ADM38061.1 (95, 100) | AEP86899.1 (93, 100) |
| BstF (YkkA) | BAI84907.2 (98, 94) | AEP90439.1 (95, 99) | ADM37387.1 (89, 100) | AEP86272.1 (92, 100) |
| BstG (DinB) | BAI84074.2 (99, 100) | AEP89654.1 (92, 100) | ADM36630.1 (88, 100) | AEP85507.1 (88, 100) |
| BstH (YizA) |  |  | ADM37148.1 (86, 100) | AEP86040.1 (85, 100) |
|  |  |  |  | AEP87498.1 |
| Homologs are listed by locus tag and were identified by Superfamily analysis of the individual genomes with *B. subtilis* BstA as the input sequence. Numbers in parenthesis represent (% sequence ID, % sequence coverage) compared to *Bacillus subtilis* 168. The protein listed in gray does not share homology with any of the *Bacillus subtilis* 168 STL proteins but were identified by the Superfamily analysis as being members of the STL family. | | | | |

| **Table B: Bacillithiol transferase homologs in *Bacillus* species** | | | | | |
| --- | --- | --- | --- | --- | --- |
| ***Bacillus subtilis* 168** | ***Bacillus amyloliquefaciens subsp. plantarum* str. FZB42**  (% sequence ID, % sequence coverage) | ***Bacillus anthracis* str. Ames**  (% sequence ID, % sequence coverage) | ***Bacillus halodurans***  **C-125**  (% sequence ID, % sequence coverage) | ***Bacillus megaterium* QM B1551**  (% sequence ID, % sequence coverage) | ***Bacillus thuringiensis* str. Al Hakam**  (% sequence ID, % sequence coverage) |
| BstA | ABS73234.1 (73, 96) | BA_2700 (53, 97)  BA_2721 (21, 80) | BAB03996.1 (38, 91) BAB04037.1 (36, 15) | ADE70783.1 (49, 95) | ABK85720.1 (52, 98)  ABK85737.1 (25, 24) |
| BstB | ABS75145.1 (71, 92) | BA_1354 (32, 98) | BAB03905.1 (27, 85)  BAB05720.1 (25, 87) | ADE67414.1 (36, 93) | ABK85564.1 (32, 94)  ABK84552.1 (32, 98) |
| BstC | ABS73463.1 (46, 96)  ABS74106.1 (38, 100) |  |  |  |  |
| BstD | ABS73607.1 (78, 99) |  |  | ADE70062.1 (59, 96)  ADE70633.1 (32, 61) |  |
| BstE | ABS74774.1 (92, 99) | BA_4768 (61, 100) | BAB05609.1 (40, 97) | ADE68689.1 (44, 100) | ABK87326.1 (63, 100) |
| BstF | ABS72955.1 (52, 85) | BA_2937 (42, 95) | BAB04544.1 (47, 88) |  | ABK85909.1, 42, 96) |
| BstG | ABS76093.1 (64, 97) | BA_2379 (44, 98) | BAB06834.1 (45, 97) | ADE70727.1 (46, 96)  ADE72658.1 (50, 99) | ABK85424.1 (42, 99) |
| BstH | ABS72600.1 (49, 94) | BA_2558 (58, 96) | BAB05611.1 (49, 94)  BAB07404.1 (25, 93) | ADE69767.1 (58, 96) | ABK85597.1 (58, 100) |
|  | ABS72947.1 | BA_2007 | BAB04718.1 | ADE68724.1 | ABK84330.1 |
|  |  | BA_2065 | BAB05333.1 | ADE68885.1 | ABK85100.1 |
|  |  | BA_2078 | BAB05771.1 | ADE69223.1 | ABK85145.1 |
|  |  | BA_2777 | BAB07706.1 | ADE69470.1 | ABK85168.1 |
|  |  | BA_2990 |  | ADE70764.1 | ABK85782.1 |
|  |  | BA_3104 |  | ADE70860.1 | ABK85840.1 |
|  |  | BA_3538 |  |  | ABK85957.1 |
|  |  | BA_3539 |  |  | ABK86047.1 |
|  |  |  |  |  | ABK86386.1 |
|  |  |  |  |  | ABK86387.1 |
|  |  |  |  |  | ABK86743.1 |
| Homologs are listed by locus tag and were identified by Superfamily analysis of the individual genomes with *B. subtilis* BstA as the input sequence. Numbers in parenthesis represent (% sequence ID, % sequence coverage) compared to *B. subtilis* 168. Proteins listed in gray do not share homology with any of the *B. subtilis* 168 STL proteins but were identified by the Superfamily analysis as being members of the STL family. | | | | | |

| **Table C: Proteomic sequence analysis of the *B. subtilis* bacillithiol transferases** | |
| --- | --- |
| ***B. subtilis* BST** | **Proteomic analysis of recombinant BSTs**  **(% sequence coverage)** |
| BstA (YfiT) | 79% |
| BstB (YuaE) | 80% |
| BstC (YisT) | 97% |
| BstD (YjoA) | 87% |
| BstE (YrdA) | 91% |
| BstF (YkkA) | 98% |
| BstG (DinB) | 96% |
| BstH (YizA) | 99% |
| Gel bands were excised from Coomassie stained SDS PAGE gels. Sequence coverage values represent peptides with >95% confidence. | |

| **Table D: Minimum inhibitory concentration (MIC) values for *B. subtilis* strains against various stressors** | | | |
| --- | --- | --- | --- |
|  | **Strain** | | |
| **Stressor** | **PY79** | **Δ*bshC*** | **Δ*bstA-H*** |
| Paraquat | 0.025 | 0.025 | 0.025 |
| CdCl_2_ | 0.025 | 0.025 | 0.025 |
| CuCl | 2.4 | 2.4 | 2.4 |
| Fosfomycin | 500 | 8 | 500 |
| NaCl | 1250 | 1250 | 1250 |
| NaOCl | 0.5 | 0.5 | 0.5 |
| Paraquat | 0.05 | 0.05 | 0.05 |
| CdCl_2_ | 0.1 | 0.1 | 0.1 |
| CuCl | >4.8 | >4.8 | >4.8 |
| Fosfomycin | 500 | 8 | 500 |
| NaCl | 1250 | 1250 | 1250 |
| NaOCl | >2.0 | >2.0 | >2.0 |
| MIC values are expressed in mM except for fosfomycin, which is expressed in μg/mL. Black text: Spizizen’s minimal media supplemented with 0.05% casamino acids and 1x trace metals solution; grey text: LB media. Growth inhibition was surveyed 24 hours after inoculation of cells. | | | |

| **Table E: Spore titers for *B. subtilis* strains against various stressors** | | |
| --- | --- | --- |
| Stressor | PY79 | Δ*bshC* |
| NaOCl^a^ | 5.0 x 10^7^ | 4.8 x 10^7^ |
| H_2_O_2_^b^ | 1.5 x 10^8^ | 1.2 x 10^8^ |
| Lysozyme^c^ | 3.1 x 10^8^ | 2.7 x 10^8^ |
| Ethanol^c^ | 2.7 x 10^8^ | 2.8 x 10^8^ |
| Methanol^c^ | 2.5 x 10^8^ | 3.2 x 10^8^ |
| Toluene^c^ | 2.3 x 10^8^ | 2.9 x 10^8^ |
| Glutaraldehyde^d^ | 2.4 x 10^8^ | 2.1 x 10^8^ |
| ^a^A suspension of spores at OD_600_=1.0 were treated with 150 μM NaOCl in 50 mM KPO_4_ buffer for 10 minutes and quenched by diluting the sample into 1% sodium thiosulfate solution as previously described[[2](#_ENREF_2)].  ^b^A suspension of spores at OD_600_=1.0 were treated with 4 M H_2_O_2_ in 50 mM KPO_4_ buffer for 10 minutes. H_2_O_2_ was inactivated by diluting the sample into 5000 U bovine catalase as previously described[[3](#_ENREF_3)].  ^c^Assays performed as previously described using OD_600_=1.0 spores[[4](#_ENREF_4)].  ^d^1 mL of a suspension of spores was diluted to a final OD_600_=1.0 in 9 mL of 2% glutaraldehyde (pH=8.0) for 10 minutes. 1 mL of the reaction was quenched in 9 mL of 2% glycine as previously described[[5](#_ENREF_5)]. | | |

| **Table F: List of plasmids used in this study** | | |
| --- | --- | --- |
| **Strain** | **Description** | **Reference or source** |
| pJLG38 | *sfGFPΩloxP-kan-loxP* | Shin and Lopez-Garrido, et al. [[6](#_ENREF_6)] |
| pRP81 | *ΔbshC*::*cat* | This study |
| pRP43 | *amyE*::*P_yizA_*-*sfGFPΩspec* | This study |
| pRP49 | *ΔdinB*::*loxP-cat-loxP* | This study |
| pRP50 | *ΔyizA*::*loxP-erm-loxP* | This study |
| pRP53 | *ΔyrdA*::*loxP-tet-loxP* | This study |
| pRP73 | *ΔyuaE*::*loxP-kan-loxP* | This study |
| pRP54 | *ΔyfiT*::*loxP-kan-loxP* | This study |
| pRP181 | *ΔykkA*::*loxP-kan-loxP* | This study |
| pRP149 | *ΔyisT*::*loxP*-*cat-loxP* | This study |
| pRP223 | *ΔyjoA*::*loxP-spec-loxP* | This study |
| pRP225 | *sacAΩtet* | This study |
| pRP260 | *ΔytxJ::loxP*-*spec-loxP* | This study |

| **Table G: List of strains used in this study** | | |
| --- | --- | --- |
| **Strain** | **Genotype or description** | **Reference, source or construction** |
| PY79 | Prototrophic derivative of *B. subtilis* 168 | Youngman et al., 1984 [[7](#_ENREF_7)] |
| NCIB 3610 | Prototroph, undomesticated parent of *B. subtilis* 168 | Branda et al., 2001 [[8](#_ENREF_8)] |
| KP1302 | NCIB 3610 lacking pBS32 | Konkol et al., 2013 [[9](#_ENREF_9)] |
| BER657 | *ΔsigH::kan* | Lab stock |
| HB11002 | *ΔbshA::mls* | Gaballa et al., 2010 [[10](#_ENREF_10)] |
| AD3303^a^ | *ΔbshA::mls* | HB11002 → PY79, Lab stock |
| EBS42 | *sacA::P_spoIIR_-creΩspec* | Becker et al., 2006 [[11](#_ENREF_11)] |
| KP648 | *Δspo0A::erm* | Jaacks et al.,1989 [[12](#_ENREF_12)] |
| RP82 | *ΔbshC*::*cat*, PY79 background | pRP81 → NCIB 3610 |
| RP148^a^ | *ΔbshC*::*cat*, 3610 background | RP82 |
| RP36 | *amyE*::*P_yfiT_*-*sfGFPΩspec*, KP1302 background | This study |
| RP24 | *amyE*::*P_yuaE_*-*sfGFPΩspec*, KP1302 background | This study |
| RP35 | *amyE*::*P_yisT_*-*sfGFPΩspec*, KP1302 background | This study |
| RP37 | *amyE*::*P_yjoA_*-*sfGFPΩspec*, KP1302 background | This study |
| RP31 | *amyE*::*P_yrdA_*-*sfGFPΩspec*, KP1302 background | This study |
| RP23 | *amyE*::*P_ykkA_*-*sfGFPΩspec*, KP1302 background | This study |
| RP28 | *amyE*::*P_dinB_*-*sfGFPΩspec*, KP1302 background | This study |
| RP98 | *amyE*::*P_yfiT_*-*sfGFPΩspec* | RP36 → PY79 |
| RP84 | *amyE*::*P_yuaE_*-*sfGFPΩspec* | RP24 → PY79 |
| RP127 | *amyE*::*P_yisT_*-*sfGFPΩspec* | RP35 → PY79 |
| RP128 | *amyE*::*P_yjoA_*-*sfGFPΩspec* | RP37 → PY79 |
| RP83 | *amyE*::*P_yrdA_*-*sfGFPΩspec* | RP31 → PY79 |
| RP97 | *amyE*::*P_ykkA_*-*sfGFPΩspec* | RP23 → PY79 |
| RP118 | *amyE*::*P_dinB_*-*sfGFPΩspec* | RP28 → PY79 |
| RP117 | *amyE*::*P_yizA_*-*sfGFPΩspec* | pRP43 → PY79 |
| RP238 | *ΔyisT*::*loxP*-*cat-loxP*, *ΔyjoA*::*loxP-spec-loxP*, *ΔdinB*::*loxP,* *ΔyizA*::*loxP, ΔyrdA*::*loxP, ΔyuaE*::*loxP, ΔyfiT*::*loxP, ΔykkA*::*loxP, sacAΩtet* | This study |
| RP259 | *ΔbrxA::loxP*-*cat-loxP, ΔbrxB::loxP*-*erm-loxP, ΔytxJ::loxP*-*spec-loxP* | This study |
| Plasmid or genomic DNA employed (right side of arrow) to transform an existing strain (left side the arrow) to create the new strain listed.  ^a^Constructed using phage-mediated transduction | | |

| **Table H: List of oligonucleotides used in this study** | |
| --- | --- |
| **Description** | **Sequence** |
| **Bacillithiol transferase promoter fusions** | |
| pDG1730 vector | F GAATTCCTGCAGCCCTGGCGAATGGC  R ATTGGAAATCGCGGCATAATC |
| *sfGFP* | F GCTAAAGGCGAAGAACTGTT  R GATTATGCCGCGATTTCCAAT |
| *yuaE* promoter region | F GCCATTCGCCAGGGCTGCAGGAATTCAGGATGCAAGATAAAAGCGC  R AACAGTTCTTCGCCTTTAGCAAAGATACTCATCATATCTC |
| *ykkA* promoter region | F GCCATTCGCCAGGGCTGCAGGAATTCACTGGATGGCGAAATCCTTG  R AACAGTTCTTCGCCTTTAGCTAAGTCCTTCATGTTCAGTT |
| *yfiT* promoter region | F GCCATTCGCCAGGGCTGCAGGAATTCTCCTTGATAGAAGTAAGTGC  R AACAGTTCTTCGCCTTTAGCGACTGATGTCATGAATGTTC |
| *dinB* promoter region | F GCCATTCGCCAGGGCTGCAGGAATTCCTCTTATGTACATTTTGGTA  R AACAGTTCTTCGCCTTTAGCAAAATCTGACATGTTAAAAT |
| *yizA* promoter region | F GCCATTCGCCAGGGCTGCAGGAATTCTCAGTATCGGCAGCAGCATC  R AACAGTTCTTCGCCTTTAGCAAATTTCATCACTAAACTCA |
| *yisT* promoter region | F GCCATTCGCCAGGGCTGCAGGAATTCACAATCTCTCTTATGATTGA  R AACAGTTCTTCGCCTTTAGCTGCATCAGTCATCAGCATGC |
| *yrdA* promoter region | F GCCATTCGCCAGGGCTGCAGGAATTCAGCAGGAAATGATGGACATT  R AACAGTTCTTCGCCTTTAGCGGTTTGAAACACAAAAATCC |
| *yjoA* promoter region | F GCCATTCGCCAGGGCTGCAGGAATTCGATCAATTGATCTGTATATC  R AACAGTTCTTCGCCTTTAGCGGATTGGCACATTATGATCT |
| **Deletion strains** | |
| Used to replace the kanamycin marker of pJLG38 with chloramphenicol | F CATTTGAGGTGATAGGTAAGCGGCAATAGTTACCCTTATTA  R GGGACCCCTATCTAGCGAACCGAAGTGGCGAGCCCGATCTT |
| Used to replace the kanamycin marker of pJLG38 with erythromycin | F CATTTGAGGTGATAGGTAAGTACTGCAATCGGATGCGATTATTG  R GGGACCCCTATCTAGCGAACATTTAGGTGTCACAAGACAC |
| Used to replace the kanamycin marker of pJLG38 with tetracycline | F CATTTGAGGTGATAGGTAAGTATTGTTGTATAAGTGATGAA  R GGGACCCCTATCTAGCGAACCCTTTCGTCTTCAAGAATTC |
| Used to replace the kanamycin marker of pJLG38 with spectinomycin | F CATTTGAGGTGATAGGTAAGGTAACGTGACTGGCAAGAGAT  R GGGACCCCTATCTAGCGAACCCTATGCAAGGGTTTATTGTT |
| Backbone for all deletion constructs amplified from pJLG38 | F GCACTTTTCGGGGAAATGTG  R GATGCATATGATCAGATCTTA |
| Antibiotic marker for all deletion constructs (used for cat, spec, erm, and tet) | F ATGAGAGAGGAAGAAAACGG  R AATTGGGACAACTCCAGTG |
| Kanamycin marker for *yuaE*, *yfiT*, and *ykkA* deletions | F AAATGAGAGAGGAAGAAAACGG  R AAAGTTCTTCTCCTTTACTA |
| *yfiT* upstream region | F TAGTAAAGGAGAAGAACTTTGACTGATGTCATGAATGTTCTC  R CACATTTCCCCGAAAAGTGCTCCTTGATAGAAGTAAGTGCTG |
| *yfiT* downstream region | F CATGATAAAGTCGAAATCTGCTATCCCGTCATATGTCGAACAG  R CCGTTTTCTTCCTCTCTCATTTAGACGGATGGGGTGGTCTTAA |
| *yuaE* upstream region | F TAGTAAAGGAGAAGAACTTTAAAGATACTCATCATATCTCC  R CACATTTCCCCGAAAAGTGCCATTATAATCGGCATTGTCAT |
| *yuaE* downstream region | F CATGATAAAGTCGAAATCTGCTGTCTAAAGAAGCAATGAAAG  R CCGTTTTCTTCCTCTCTCATTTAGATAGAAAGGGCCTGATTTA |
| *yisT* upstream region | F CATGATAAAGTCGAAATCTGCACATTATACATTTGGCTGGTTC  R CCGTTTTCTTCCTCTCTCATTGCATCAGTCATCAGCATGCC |
| *yisT* downstream region | F CACTGGAGTTGTCCCAATTAAAAGAGAAAGCAAGGGCTAA  R CACATTTCCCCGAAAAGTGCCACACATGGTGATATGATCATC |
| *yjoA* upstream region | F CATGATAAAGTCGAAATCTGCTCGAATCTATATCCTCGATGAC  R CCGTTTTCTTCCTCTCTCATGGATTGGCACATTATGATCTC |
| *yjoA* downstream region | F CACTGGAGTTGTCCCAATTTTCTATCAGCAGCGCATGTAG  R CACATTTCCCCGAAAAGTGCCCGCTGACTAAAATAATGGAAGCC |
| *yrdA* upstream region | F CATGATAAAGTCGAAATCTGCATGCGAAGTGAGCAGGAAATGA  R CCGTTTTCTTCCTCTCTCATGGTTTGAAACACAAAAATCCTC |
| *yrdA* downstream region | F CACTGGAGTTGTCCCAATTTTGGAAGCCCCGAAAATGTAA  R CACATTTCCCCGAAAAGTGCTCTCTGCTTTATTTCAGCGTA |
| *ykkA* upstream region | F TAGTAAAGGAGAAGAACTTTAAGTCCTTCATGTTCAGTTC  R CACATTTCCCCGAAAAGTGCGGGTATTTTTACTGAAGCCAG |
| *ykkA* downstream region | F CATGATAAAGTCGAAATCTGCGTCTCACATGAGATTGACTCT  R CCGTTTTCTTCCTCTCTCATTTCGAAGTATGAACGCTAAAACG |
| *dinB* upstream region | F CATGATAAAGTCGAAATCTGCACTTGAATTTGATGATTGCTC  R CCGTTTTCTTCCTCTCTCATAAAATCTGACATGTTAAAATTC |
| *dinB* downstream region | F CACTGGAGTTGTCCCAATTACAAAAACTGAAAAAGCATAA  R CACATTTCCCCGAAAAGTGCCGAGAGGATAAACCCGTGCAT |
| *yizA* upstream region | F CATGATAAAGTCGAAATCTGCCAGCCCGGTGCAAATGAAGAG  R CCGTTTTCTTCCTCTCTCATAAATTTCATCACTAAACTCACT |
| *yizA* downstream region | F CACTGGAGTTGTCCCAATTAAACCTATTCATTCTTATTGA  R CACATTTCCCCGAAAAGTGCCAAATATGAAGAAACCTTGGC |
| *brxA* upstream region | F CATGATAAAGTCGAAATCTGCGCGCTATCATTAAAACAGGCG  R CCGTTTTCTTCCTCTCTCATCATTGACATAAAAAAAGCCCCCTCTAG |
| *brxA* downstream region | F CACTGGAGTTGTCCCAATTCACTGCTAAATGCCCGTTCTC  R CACATTTCCCCGAAAAGTGCAGAGTTGTCAGGGAGCTGCTT |
| *brxB* upstream region | F CATGATAAAGTCGAAATCTGCGTTGATTTGATTCGACGTTTC  R CCGTTTTCTTCCTCTCTCATCATGTTCAATGAAGACCTCTC |
| *brxB* downstream region | F CACTGGAGTTGTCCCAATTGAAGTATAAGACGAACAACCC  R CACATTTCCCCGAAAAGTGCATATGGTTCGCTGTGTAATAC |
| *ytxJ* upstream region | F CATGATAAAGTCGAAATCTGC  R CCGTTTTCTTCCTCTCTCAT |
| *ytxJ* downstream region | F CACTGGAGTTGTCCCAATT  R CACATTTCCCCGAAAAGTGC |
| *sacA* upstream region | F CATGATAAAGTCGAAATCTGCGATATCGCTCTTCTCGGCTAC  R CTTACCTATCACCTCAAATGCTACATAAGTGTCCAAATTCC |
| *sacA* downstream region | F GTTCGCTAGATAGGGGTCCCAAAATCCTTCTATTTTCTTATG  R CACATTTCCCCGAAAAGTGCCATTCTGCACTGCGCCTGAATC |
| Tetracycline antibiotic marker for *sacAΩtet* construct | F TATTGTTGTATAAGTGATGAA  R GGGACCCCTATCTAGCGAAC |
| Homology regions for Gibson assembly are shown underlined. | |

**SUPPLEMENTARY MATERIALS AND METHODS**

**Proteomics Methods**

LC-MS-MS methods (Fig. 2A)

Trypsin-digested peptides were analyzed by HPLC coupled with tandem mass spectroscopy (LC-MS/MS) using nanospray ionization. The nanospray ionization experiments were performed using a TripleTof 5600 hybrid mass spectrometer (ABSCIEX) interfaced with nanoscale reversed-phase HPLC (Tempo) using a 10 cm-100 micron ID glass capillary packed with 5-µm C18 Zorbax^TM^ beads (Agilent Technologies, Santa Clara, CA).  Peptides were eluted from the C18 column into the mass spectrometer using a linear gradient (5–60%) of ACN (Acetonitrile) at a flow rate of 250 μl/min for 1h. The buffers used to create the ACN gradient were: Buffer A (98% H_2_O, 2% ACN, 0.2% formic acid, and 0.005% TFA) and Buffer B (100% ACN, 0.2% formic acid, and 0.005% TFA). MS/MS data were acquired in a data-dependent manner in which the MS1 data was acquired for 250 ms at m/z of 400 to 1250 Da and the MS/MS data was acquired from m/z of 50 to 2,000 Da. For Independent data acquisition (IDA) parameters MS1-TOF 250 milliseconds, followed by 50 MS2 events of 25 milliseconds each. The IDA criteria; over 200 counts threshold, charge state of plus 2-4 with 4 seconds exclusion window. Finally, the collected data were analyzed using MASCOT^®^ (Matrix Sciences) and Protein Pilot 4.0 (ABSCIEX) for peptide identifications.

Protein Digestion and TMT Labeling (Fig. 2B)

Insoluble debris was pelleted by centrifugation at 20,000 xg for 5 minutes. Proteins were precipitated with trichloroacetic acid (TCA) then re-solubilized in 1M urea (Fisher) in 50mM HEPES, pH 8.5. Proteins were digested in a two-step process; 3μg of LysC (Wako) was added to each sample and then incubated overnight at room temperature. Next, 3μg of trypsin was added and samples were digested for 6 hours at 37°C. Digestion was quenched with trifluoroacetic acid (TFA, Pierce) then desalted with C18 Sep-Paks (Waters) as previously described [[13](#_ENREF_13)].

Samples were labeled with 10-plex TMT reagents (Thermo Fisher Scientific) [[14](#_ENREF_14),[15](#_ENREF_15)] as previously described [[16](#_ENREF_16)]. TMT reagents were solubilized in dry acetonitrile (Sigma) at 20μg/μL. Peptides were re-suspended in 30% dry acetonitrile in 200mM HEPES, pH 8.5 and 7μL of the appropriate TMT reagent was added to each sample. Labeling was conducted for 1 hour at room temperature and was quenched by addition of 8μL of 5% hydroxylamine (Sigma), which was allowed to react for 15 minutes. Samples were then acidified by addition of 50μL of 1% TFA and pooled. The pooled sample was desalted with C18 Sep-Paks as described above.

*Basic pH Reverse-Phase Liquid Chromatography Fractionation*

Fractionation was carried out by basic pH reverse-phase liquid chromatography [[17](#_ENREF_17)] with fraction combining as previously described [[13](#_ENREF_13)]. Briefly, samples were solubilized in 5% formic acid in 5% acetonitrile and separated on a 4.6mm x 250mm C28 column (Thermo Fisher Scientific) on an Ultimate 3000 HPLC fitted with an auto sampler, fraction collector, degasser and variable wavelength detector. Separation was performed over a 22% to 35%, 60-minute linear gradient of acetonitrile in 10mM ammonium bicarbonate (Fisher) at a flow rate of 0.5 mL/min. The resultant 96 fractions were combined as previously described [[13](#_ENREF_13)]. Fractions were dried and re-suspended in 5% formic acid/5% acetonitrile and analyzed by LC-MS2/MS3 for identification and quantitation.

*LC-MS2/MS3 Protein Identification and Quantitation*

LC-MS2/MS3 experiments were conducted on an Orbitrap Fusion (Thermo Fisher Scientific) with an in-line Easy-nLC 1000 (Thermo Fisher Scientific). Home-pulled, home-packed columns (100μm ID x 30cm, 360μ OD) were used for analysis. Analytical columns were triple-packed with 5μm C4 resin, 3μm C18 resin and 1.8μm C18 resin (Sepax) to lengths of 0.5cm, 0.5cm and 30cm respectively. Samples were loaded on the column at 500 bar and eluted with a linear gradient of 11% to 30% acetonitrile in 0.125% formic acid over 165 minutes at a flow rate of 300nL/minute with the column heated to 60°C. Nano-electrospray ionization was achieved by applying 2000V through a stainless steel T-junction at the inlet of the analytical column.

The Orbitrap Fusion was run in data-dependent mode, where a survey scan was collected over 500-1200m/z at a resolution of 120000 in the Orbitrap. Automatic gain control (AGC) was set to 5x10^5^ for the survey scan, with a maximum ion injection time of 100ms. The S-lens RF was set to 60 and centroided data were collected. For subsequent MS2/MS3 analysis, top speed mode was enabled to select the most abundant ions for analysis in a 5 second cycle.

For MS2/MS3 analysis, the decision tree option was used, with charge state and m/z range as qualifiers. Ions with a +2 charge state were analyzed from the m/z range of 600-1200, and +3/+4 ions were selected from the m/z range of 500-1200. An ion intensity trigger threshold of 5x10^3^ was used. MS2 spectra were obtained with quadrupole isolation with a 0.5 Th window and fragmented with collision induced dissociation using a normalized collision energy of 30%. Fragment ions were detected and centroided data collected in the linear ion trap using rapid scan rate with an AGC target of 1x10^4^, maximum ion injection time of 35 ms.

MS3 analysis was conducted using the synchronous precursor selection (SPS) option to maximize TMT quantitation sensitivity [[14](#_ENREF_14)]. For SPS, a maximum of 10 MS2 precursors was specified, which were simultaneously isolated and fragmented for MS3 quantitation. Higher-energy collisional dissociation was used as fragmentation for MS3, with a normalized collision energy of 50%. Resultant fragments were detected in the Orbitrap with a resolution of 60000 and a low mass cut-off of 110m/z. AGC for MS3 spectra was set to 5x10^4^ with a maximum ion injection time of 250ms. MS2 ons from a range of 40m/z below and 15m/z above the precursor m/z were excluded by SPS. Centroided data were collected for all MS3 scans.

*Data Processing and Analysis*

Resultant data files were processed using Proteome Discoverer 2.1 (Thermo Fisher Scientific). MS2 data were queried against the Uniprot *Bacillus subtilis* database using the Sequest algorithm [[18](#_ENREF_18)]. A decoy search was also conducted with sequences in reversed order [[19-21](#_ENREF_19)]. For MS1 spectra, a mass tolerance of 50ppm was specified and for MS2 spectra a 0.6Da tolerance was used. Static modifications included TMT 10-plex reagents on lysine and peptide N-termini (+229.162932Da). Variable oxidation of methionines (+15.99492Da), bacillithiol modification of cysteines (+396.08387Da) and carbamidomethylation of cysteines (+57.02146Da) were also included in the search parameters. Data were filtered to a 1% peptide and protein level false discovery rate using the target-decoy strategy [[19](#_ENREF_19)].

Reporter ion intensities from TMT reagents were extracted from MS3 spectra for quantitative analysis, and signal to noise values were used for quantitation. Spectra were used if the average signal to noise was greater than 10 across samples and if isolation interference was less than 25%. Protein level quantitation values were calculated by summing signal to noise values for all peptides per protein meeting the specified filters. Data were normalized in a two-step process, whereby they were first normalized to the mean for each protein. To account for variation in the amount of protein labeled, values were then normalized to the median of the entire dataset. Final values are reported as normalized summed signal to noise per protein per sample.

**References:**

1. Newton GL, Leung SS, Wakabayashi JI, Rawat M, Fahey RC (2011) The DinB superfamily includes novel mycothiol, bacillithiol, and glutathione S-transferases. Biochemistry 50: 10751-10760.

2. Young SB, Setlow P (2003) Mechanisms of killing of Bacillus subtilis spores by hypochlorite and chlorine dioxide. J Appl Microbiol 95: 54-67.

3. Setlow B, Setlow P (1993) Binding of small, acid-soluble spore proteins to DNA plays a significant role in the resistance of Bacillus subtilis spores to hydrogen peroxide. Appl Environ Microbiol 59: 3418-3423.

4. Harwood CR, Cutting SM (1990) Molecular biological methods for Bacillus. Chichester ; New York: Wiley. xxxv, 581 p. p.

5. Power EGM, Dancer BN, Russell AD (1988) Emergence of resistance to glutaraldehyde in spores of Bacillus subtilis 168. FEMS Microbiol Lett 50: 223-226.

6. Yen Shin J, Lopez-Garrido J, Lee SH, Diaz-Celis C, Fleming T, et al. (2015) Visualization and functional dissection of coaxial paired SpoIIIE channels across the sporulation septum. Elife 4: e06474.

7. Youngman P, Perkins JB, Losick R (1984) Construction of a cloning site near one end of Tn917 into which foreign DNA may be inserted without affecting transposition in Bacillus subtilis or expression of the transposon-borne erm gene. Plasmid 12: 1-9.

8. Branda SS, Gonzalez-Pastor JE, Ben-Yehuda S, Losick R, Kolter R (2001) Fruiting body formation by Bacillus subtilis. Proc Natl Acad Sci U S A 98: 11621-11626.

9. Konkol MA, Blair KM, Kearns DB (2013) Plasmid-encoded ComI inhibits competence in the ancestral 3610 strain of Bacillus subtilis. J Bacteriol 195: 4085-4093.

10. Gaballa A, Newton GL, Antelmann H, Parsonage D, Upton H, et al. (2010) Biosynthesis and functions of bacillithiol, a major low-molecular-weight thiol in Bacilli. Proc Natl Acad Sci U S A 107: 6482-6486.

11. Becker E, Herrera NC, Gunderson FQ, Derman AI, Dance AL, et al. (2006) DNA segregation by the bacterial actin AlfA during Bacillus subtilis growth and development. EMBO J 25: 5919-5931.

12. Jaacks KJ, Healy J, Losick R, Grossman AD (1989) Identification and characterization of genes controlled by the sporulation-regulatory gene spo0H in Bacillus subtilis. J Bacteriol 171: 4121-4129.

13. Tolonen AC, Haas W (2014) Quantitative proteomics using reductive dimethylation for stable isotope labeling. J Vis Exp.

14. McAlister GC, Nusinow DP, Jedrychowski MP, Wuhr M, Huttlin EL, et al. (2014) MultiNotch MS3 enables accurate, sensitive, and multiplexed detection of differential expression across cancer cell line proteomes. Anal Chem 86: 7150-7158.

15. Thompson A, Schafer J, Kuhn K, Kienle S, Schwarz J, et al. (2003) Tandem mass tags: a novel quantification strategy for comparative analysis of complex protein mixtures by MS/MS. Anal Chem 75: 1895-1904.

16. Ting L, Rad R, Gygi SP, Haas W (2011) MS3 eliminates ratio distortion in isobaric multiplexed quantitative proteomics. Nat Methods 8: 937-940.

17. Wang Y, Yang F, Gritsenko MA, Wang Y, Clauss T, et al. (2011) Reversed-phase chromatography with multiple fraction concatenation strategy for proteome profiling of human MCF10A cells. Proteomics 11: 2019-2026.

18. Eng JK, McCormack AL, Yates JR (1994) An approach to correlate tandem mass spectral data of peptides with amino acid sequences in a protein database. J Am Soc Mass Spectrom 5: 976-989.

19. Elias JE, Gygi SP (2007) Target-decoy search strategy for increased confidence in large-scale protein identifications by mass spectrometry. Nature Methods 4: 207-214.

20. Elias JE, Haas W, Faherty BK, Gygi SP (2005) Comparative evaluation of mass spectrometry platforms used in large-scale proteomics investigations. Nat Methods 2: 667-675.

21. Peng J, Elias JE, Thoreen CC, Licklider LJ, Gygi SP (2003) Evaluation of multidimensional chromatography coupled with tandem mass spectrometry (LC/LC-MS/MS) for large-scale protein analysis: the yeast proteome. J Proteome Res 2: 43-50.
